# Health monitoring in birds using bio-loggers and whole blood transcriptomics

**DOI:** 10.1038/s41598-021-90212-8

**Published:** 2021-05-24

**Authors:** Elinor Jax, Inge Müller, Stefan Börno, Hanna Borlinghaus, Gustaw Eriksson, Evi Fricke, Bernd Timmermann, Helene Pendl, Wolfgang Fiedler, Karsten Klein, Falk Schreiber, Martin Wikelski, Katharine E. Magor, Robert H. S. Kraus

**Affiliations:** 1 Department of Migration, Max Planck Institute of Animal Behavior, 78315 Radolfzell, Germany; 2 Department of Biology, University of Konstanz, 78464 Konstanz, Germany; 3 Sequencing Core Facility, Max Planck Institute for Molecular Genetics, 14195 Berlin, Germany; 4 Department of Computer and Information Science, University of Konstanz, 78464 Konstanz, Germany; 5 Department of Biology, Lund University, 223 62 Lund, Sweden; 6 Pendl Lab, 6300 Zug, Switzerland; 7 Faculty of Information Technology, Monash University, Clayton, VIC 3800 Australia; 8 Centre for the Advanced Study of Collective Behaviour, University of Konstanz, 78464 Konstanz, Germany; 9 Department of Biological Sciences and Li Ka Shing Institute of Virology, University of Alberta, Edmonton, AB T6G 2R3 Canada

**Keywords:** RNA sequencing, Gene ontology, Gene regulatory networks, High-throughput screening, Infectious diseases, Innate immune cells, High-throughput screening, Gene expression, Immunology, Non-model organisms

## Abstract

Monitoring and early detection of emerging infectious diseases in wild animals is of crucial global importance, yet reliable ways to measure immune status and responses are lacking for animals in the wild. Here we assess the usefulness of bio-loggers for detecting disease outbreaks in free-living birds and confirm detailed responses using leukocyte composition and large-scale transcriptomics. We simulated natural infections by viral and bacterial pathogens in captive mallards (*Anas platyrhynchos*), an important natural vector for avian influenza virus. We show that body temperature, heart rate and leukocyte composition change reliably during an acute phase immune response. Using genome-wide gene expression profiling of whole blood across time points we confirm that immunostimulants activate pathogen-specific gene regulatory networks. By reporting immune response related changes in physiological and behavioural traits that can be studied in free-ranging populations, we provide baseline information with importance to the global monitoring of zoonotic diseases.

## Introduction

Wild animals are important reservoirs of a wide range of infectious diseases, of which some have the potential to spill over to humans (zoonoses)^1^. Major modern human diseases such as influenza and salmonellosis are frequently of zoonotic origin^2^. While zoonoses may have major impact on human health, the natural hosts of these diseases often show little signs of disease upon infection^3^. This allows for these diseases to persist in reservoir species with a risk of interspecies transmission to more susceptible host-species. Reservoir host physiology and behaviour during infection affect survival and disease duration in the host^4^ and therefore also affect the spread of infectious diseases^5^. Still little is known about the immune response of reservoir hosts in their natural environment^3^. Several hurdles have constrained our understanding of pathogen dynamics and immune responses in wild reservoir hosts. These include, but are not limited to (1) the scarce availability of toolsets available for measuring immune responses in free-ranging non-model species in comparison to those available for well-studied model species^6,7^, and (2) lack of detailed experimental data from many reservoir species making it difficult to interpret the results from immunological tests in a field setting^3,7^.

The innate immune system provides the first line of defence against pathogens. An important part of the innate immune system is the acute-phase response (APR), a rapid and systemic response activated by trauma, inflammation, stress and infection^8^. The APR is triggered by the release of proinflammatory cytokines in immune cells, and is characterised by a suite of molecular, physiological, and behavioural changes^9,10^. Animals with an activated APR usually have a febrile response within hours of pathogen exposure^11^, but the duration and magnitude of the febrile response differ between host species^12–14^ and depend on the type of stimuli^14–16^. Besides fever, animals usually display sickness behaviours such as lethargy, depression and anorexia during the APR^9,10^. The APR has an important role for the recovery and survival of the host during infection^4,17^, and may thus affect the risk of further transmission of infectious agents^5,17^. Learning about the APR in reservoir species is therefore important for understanding their potential role in the spread of zoonotic diseases, and monitoring their APR in the field could be used as a warning system for disease outbreaks.

Rapid advancement of bio-logging technologies now allows for the study of physiology and behaviour of free-living animals^18^. Bio-loggers that can detect changes in important characteristics of the APR (including body temperature, movement patterns and energy expenditure) are increasingly being used in wildlife research^19^, and thus provide information that could be used for identifying signs and symptoms of disease in reservoir species. Similarly, rapid advances in and decreased costs of next generation DNA and RNA sequencing technologies now allow researchers to study the underlying mechanisms of the APR in non-model species, and provides disease markers for studying immune status and responses in wild populations^20–22^. While these technological advances have been used to study changes in behaviour^23^, physiology^24^ and regulation of immune genes in free-living animals^25^, they have so far not been studied simultaneously during the APR in reservoir species of zoonotic diseases. Studies that define the baseline for these measurements and their deviation during the immune response in reservoir species will provide a valuable resource for disease monitoring in natural populations.

The aim of this study was to identify reliable and repeatable, integrated multi-scale monitoring methods from several biological fields that will facilitate ecological immunology studies and disease monitoring in wild bird reservoirs. Further we wanted to identify known^20,26^ and novel candidate genes that are upregulated during different stages of the APR and in response to different pathogens, in tissues that can easily and repeatably be collected in free-living birds. For this purpose, we experimentally induced immune responses in an important natural vector for avian influenza virus (AIV), the mallard (*Anas platyrhynchos*)^27,28^. While mallards show few signs of disease when infected with low pathogenic strains of AIV^29^, they can show clinical signs and symptoms including fever, anorexia, neurological signs and death when infected with highly pathogenetic AIV^30,31^. To study the APR in mallards, we triggered the immune response in captive mallards using non-infectious immunostimulants and monitored the response with state-of-the-art bio-loggers measuring 3D acceleration, heart rate, and body temperature and with high throughput RNA sequencing technologies. Our specific aims were to (1) examine the magnitude and timing of changes in the bio-logged parameters, (2) assess whether a correspondent immune response can be detected in global gene expression profiles from whole blood, and (3) determine the specificity of the immune response to different stimulants, using transcriptomics and gene regulatory network analyses.

## Results

### Body temperature, heart rate and activity

We challenged mallards with three types of non-infectious immuno-stimulants, thereby mimicking natural infections by RNA viruses (polyinosinic:polycytidylic acid, poly I:C), gram-negative bacteria (lipopolysaccharide, LPS) and gram-positive bacteria (cell walls of heat-killed *Staphylococcus aureus*). To determine if the immunostimulants caused physiological and behavioural changes in the mallards, we monitored changes in body temperature, heart rate and activity in three individuals per treatment group using bio-loggers (Supplementary Information Figure S1). We fitted generalised additive mixed models (GAMMs) to the data until 18.5 h post stimulation (hps) for each physiological measurement (Supplementary Information Tables S1–S3) to compare the effect of the different treatments.

**Figure 1:**
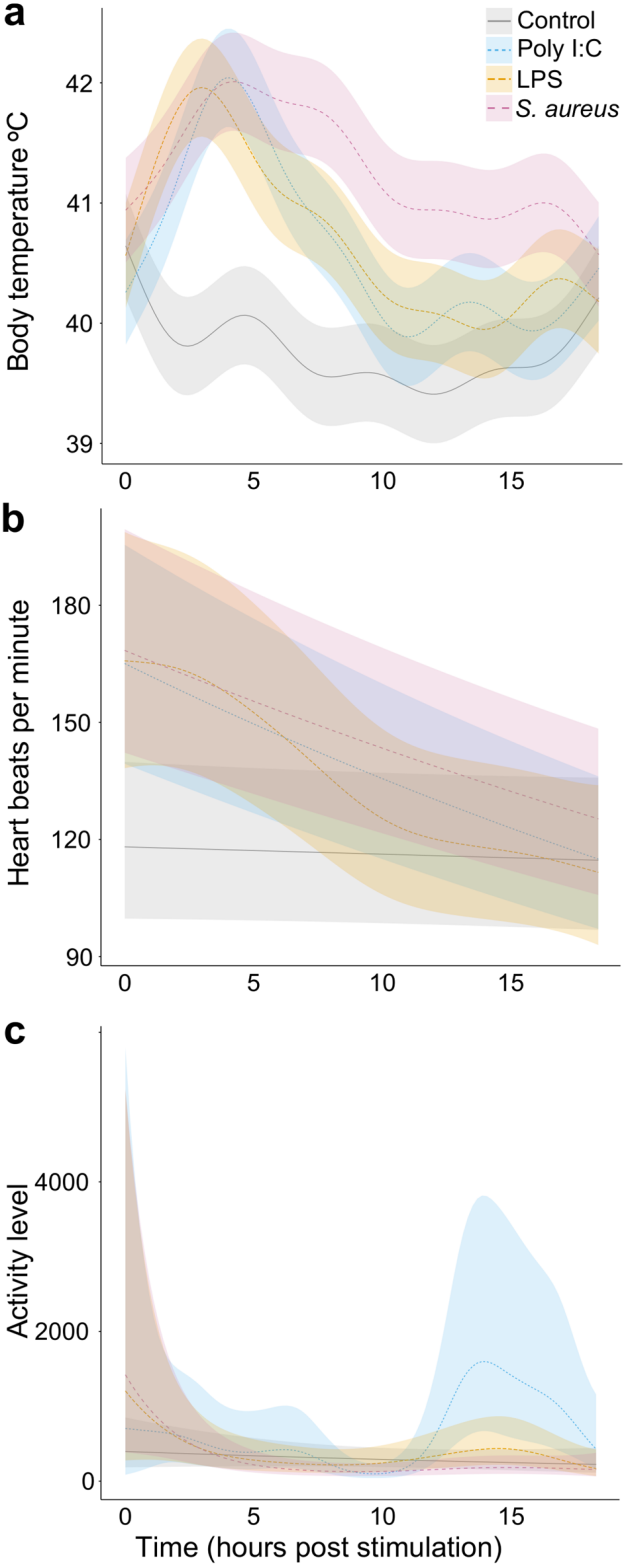
Physiological changes following challenge with immune stimulants. Changes in (**a**) body temperature, (**b**) heart rate and (**c**) activity level were measured remotely using bio-loggers. Mean and 95% credible interval for each treatment group was plotted until 18.5 h post stimulation, as estimated from the posterior distribution of the GAMM. An activity value of 0 means no activity, while higher values mean more movement in either or all of the three axes.

To track the timing of the febrile response, we measured body temperature after administration of immune stimulants. The average body temperature increased in all treatment groups when compared to the control group (Fig. 1a). The maximum mean temperature in the poly I:C group was reached after 4.1 hps (n = 3, mean 42.04 °C, 95% Credible Intervals (CrI) 41.63–42.45 °C) and was elevated until 10.5 hps. The maximum mean temperature in the LPS group was reached after 3.0 hps (n = 3, mean 41.96 °C, CrI 41.55–42.37 °C), and was elevated until 14.9 hps. The maximum mean temperature in the *S. aureus* group was reached after 4.2 hps (n = 3, mean 42.01 °C, CrI 41.60–42.42 °C) and was elevated until 18.5 hps—compared to ~ 40–41 °C in the control group.

To assess the stress response, we examined the heart rate in the mallards following stimulation. The average heart rate was elevated in all treatment groups immediately following the stimulation (Fig. 1b). The heart rate remained higher in the stimulated groups than in the control group until 8.8 hps for the poly I:C group, until 7.8 hps for the LPS group, and until 13.8 hps for the *S. aureus* group.

Inactivity is often a sign of disease, so we monitored activity levels using high definition accelerometers that record acceleration along three axes^32^. No clear differences were apparent during the response to treatment, however, ducks showed increased activity upon recovery from the viral mimic (Fig. 1c).

### Leukocyte differential count

**Figure 2:**
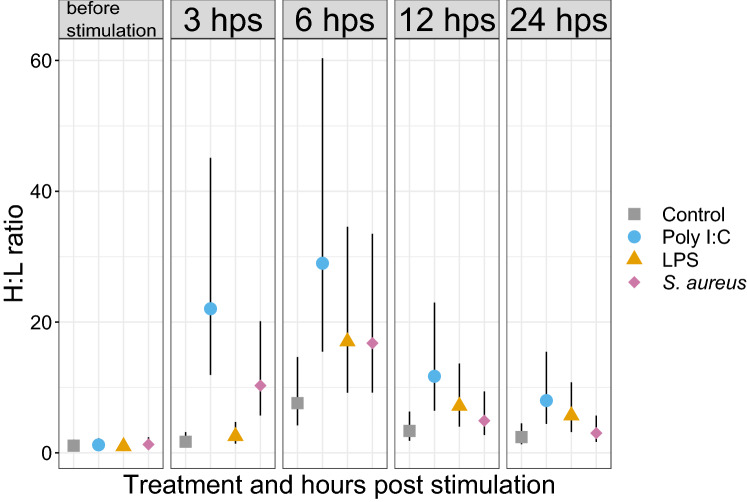
Elevated H:L ratios in mallard blood following challenge with immune stimulants. Mean H:L ratio and 95% credible intervals as estimated from the posterior distribution from the multinomial model (n = 5 ducks/timepoint).

Acute phase response to disease is often accompanied by the recruitment of neutrophils into circulating blood resulting in a blood neutrophilia. Like neutrophils, their counterpart in birds, heterophils, are critically involved in the immediate response to pathogens^33^. To confirm that changes in blood leukocyte composition accompanied immune responses to stimulants in our study, we estimated the mean leukocyte proportions (Supplementary Information Text S1 and Figure S2) and the heterophil:lymphocyte (H:L) ratio for each treatment group. We performed differential leukocyte counts on more than 200 cells on stained blood films using light microscopy for five individuals per treatment group at each time point. We observed an increase in the H:L ratio, with a peak at 6 hps, for all treatment groups (Fig. 2).

### Genome wide gene expression profiling

#### RNA-sequencing and differential gene expression

**Figure 3:**
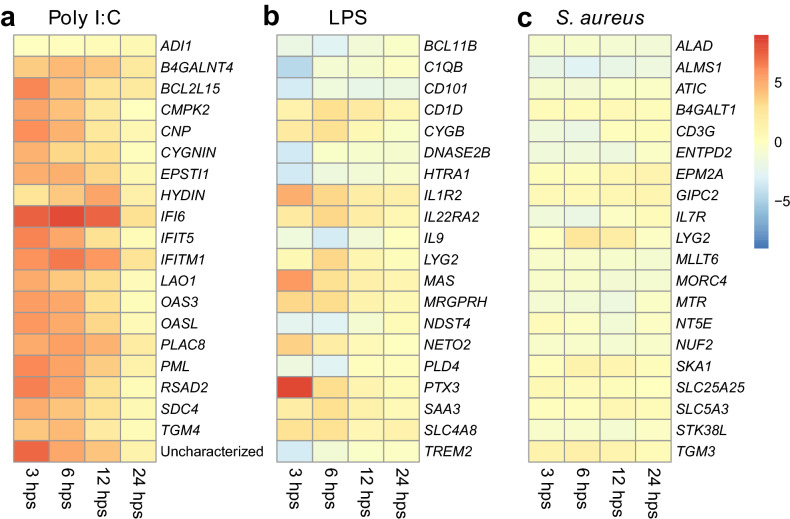
Heatmap illustrating the log2 fold change of the differentially expressed genes (rows) that were most up- or downregulated for each time point (column) and treatment group (FDR < 0.05). Red indicates that the gene expression was higher-, and blue indicates that the gene expression was lower in the treatment group than in the control group. For genes that could not be assigned a gene name from the mallard genome, hits identified through the BLAST search is shown (for more details see Supplementary Information Tables S4–S7 and Supplementary Dataset S4. Gene name changed from IFITM3 to IFITM1, following the suggested nomenclature in Blyth, et al.^61^. All DEGs from the treatment groups are listed in Supplementary Datasets S1–S3.

To identify differentially expressed genes (DEGs) following administration of immune stimulants, we performed full transcriptome sequencing on blood samples from three females and three males for each treatment and timepoint, adding up to a total of 120 samples (Supplementary Information Text S2). As our preliminary analyses did not detect a clear difference in the response between females and males, we included individuals from both sexes in the differential expression analyses (Supplementary Information Text S3). The number of DEGs for each treatment group peaked at 1016 following poly I:C challenge, 256 for the LPS challenge and 94 for the *S. aureus* challenge, however, this differed between the time-points (Supplementary Datasets S1–S3, Supplementary Information Figure S3). In the poly I:C and the LPS treatment group the majority of the genes were differentially expressed at 3 and 6 hps, indicating a rapid response to the treatments. In contrast, the majority of DEGs in the *S. aureus* treatment group were detected at 12 hps. To identify key genes that can be used to assess immune status in a field experiment, we identified the top DEGs from each time point and each treatment (Fig. 3, Supplementary Information Tables S4–S6, Text S4). The overlap of significantly DEGs between the different treatment groups was moderate, with roughly 13.6%, 7.5%, 4%, and 0% of the DEGs in any of the treatment groups being shared between two or more treatment groups at time points 3 h, 6 h, 12 h and 24 hps respectively (Supplementary Information Figure S4, Text S5). Thus, the differential gene expression analyses suggest that the immune response to each immunostimulant is unique.

#### Gene ontology (GO) analysis and enrichment test

**Figure 4:**
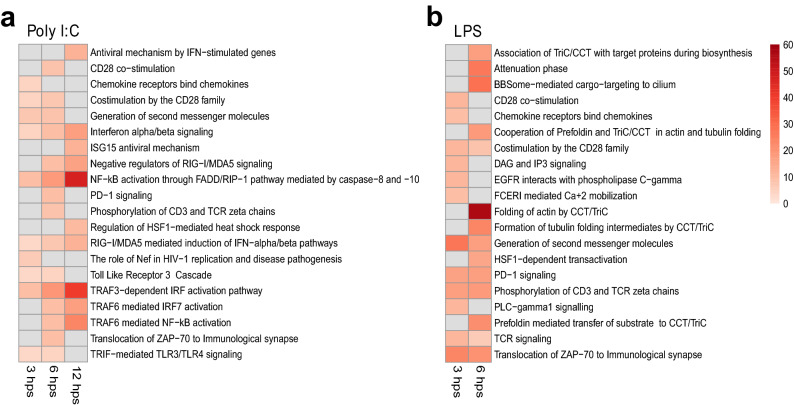
Heatmap illustrating overrepresented Reactome Pathways with the highest fold enrichment score for the poly I:C and LPS treatment groups. Pathways that were significantly overrepresented (FDR < 0.05) are shown in red colour, with faint red indicating lower fold enrichment score and dark red a higher fold enrichment score. Pathways that were not significantly overrepresented are shown in grey for that particular treatment group and time point. No Reactome Pathways were overrepresented in the *S. aureus* treatment group, nor 24 hps in the poly I:C treatment group or 12/24 hps in the LPS treatment group. All overrepresented gene ontology terms are listed in Supplementary Datasets S5–S6.

We performed a gene ontology (GO) analysis to investigate whether certain biological processes and pathways were overrepresented in our list of DEGs. We found a significant overrepresentation of Reactome pathways in the poly I:C treatment group at 3 (n = 25), 6 (n = 29), and 12 h (n = 14) ps and in the LPS treatment group 3 (n = 62) and 6 hps (n = 29) (Supplementary Datasets S5–S6). In the poly I:C treatment group, overrepresented Reactome pathways were found within functions such as antiviral responses, adaptive and innate immune system, and interferon signaling (Fig. 4 and Supplementary Dataset S5, Supplementary Information Text S6). Several of the overrepresented Reactome pathways in the LPS treatment group were related to T-cell activation and signaling, adaptive immune system functions and heat shock and stress responses (Fig. 4 and Supplementary Dataset S6, Supplementary Information Text S6). No overrepresented pathways were detected for the *S. aureus* treatment group. The overrepresented Reactome Pathways with the highest enrichment score for each of these groups are shown in Fig. 4. The results from the GO overrepresentation analysis for the Biological Processes (Supplementary Information Figure S5, Text S7) were similar to those from the GO overrepresentation analysis for the Reactome pathway.

#### Data mapping onto KEGG pathways

To further explore the specificity of the immune response to each immunostimulant, we visualised the gene expression profiles for each treatment and time point in the context of gene networks. To facilitate exploration of the results as well as future comparative studies, we built an interactive webpage (http://orn-files.iwww.mpg.de/dgeviz) where the gene expression fold changes are illustrated on seven immune related pathways from the Kyoto Encyclopedia of Genes and Genomes (KEGG) database^34–36^. The fold changes and corrected *p* values for all treatments and pathways are available in Supplementary Dataset S7.

**Figure 5:**
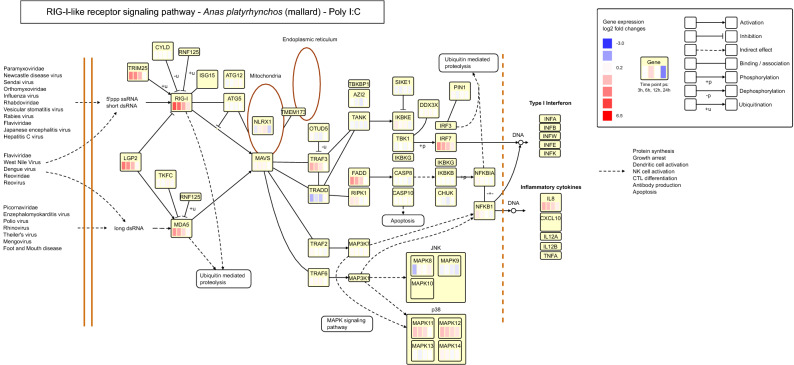
Log2 fold changes of the expression level between the poly I:C and control treatments mapped on the RIG-I like receptor signaling pathway from the KEGG database^34–36^. Red indicates that the gene expression was higher, and blue indicates that the gene expression was lower in the poly I:C treatment group than in the control group. White indicates no change or a similar change in the poly I:C treatment group as in the control group. Each box represents one gene in the pathway and the columns within the box show the gene expression fold change for the four time points; 3, 6, 12 and 24 hps from left to right. For more details see http://orn-files.iwww.mpg.de/dgeviz/RIG_PICButton.html.

The Reactome pathways showing highest differential gene expression following poly I:C stimulation included RIG-like receptor, TLR3, and interferon-alpha signaling pathways, as expected. Within the RIG-I/MDA5 signaling pathway several genes were significantly upregulated at one or several of the time points following the poly I:C treatment (Fig. 5, Supplementary Dataset S1). Most notably, *RIG-I* (Retinoic acid-Inducible Gene I; also called *DDX58*) and *MDA5* (Melanoma Differentiation-Associated protein 5; also called *IFIH1*) were upregulated at early time points, as were the activating protein *TRIM25* (Tripartite Motif Containing 25), and downstream signaling molecules *TRAF* (TNF Receptor Associated Factor) and *FADD* (Fas Associated Via Death Domain), and interferon regulatory protein *IRF7* (Interferon Regulatory Factor 7). Details of the toll-like receptor signaling pathway and the Influenza A pathway are shown and discussed in Supplementary Information (Figures S6–S7, Text S8).

#### Gene expression of target genes

We measured gene expression of five key immune genes, identified in our transcriptome analysis for the poly I:C treatment group, from nine individuals using real-time qPCR to validate our RNA-seq results. The gene expression results from the real-time qPCR confirmed the RNA-seq results (Supplementary Information Figures S8–S9). Of the five measured genes, *RSAD2* was the most highly expressed gene, followed by *RIG-I*, *TLR3* and *IRF7* (Supplementary Information Figure S8). Finally, the genes mined from our transcriptome analyses provide a panel of target genes for analysis of antiviral responses in mallards.

## Discussion

We quantified several important physiological traits in healthy and immune-challenged individuals of birds in a controlled setup, and thereby provide crucial information that can be used for disease monitoring in wild populations. By combining state-of-the-art bio-loggers to monitor an immune response in progress with high throughput sequencing technologies to characterise genome wide gene expression profiles in blood samples collected across several timepoints, we were able to examine the duration and magnitude of the immune response in relation to each immune-stimulant.

An acute phase response includes physiological and behavioural changes. Studying such changes during infection in animals is not trivial, and usually includes disturbance of the animals for sampling or observation. One of the main goals of this study was to investigate if the acute phase response (APR) can be monitored using on-animal bio-loggers, technologies that have great applications for remote, long-term disease monitoring in wild populations. For this purpose, we recorded changes in body temperature, heart rate and activity in control and immune challenged mallards using bio-loggers, while simultaneously monitoring the cellular response using blood immune assays. Elevated body temperature can be used to quantify the extent of a fever response in birds^37^. Heart rate is linked to oxygen consumption via Fick’s equation and is often used as a means of estimating energy expenditure in (wild) animals^38,39^. High definition accelerometers that record acceleration along three axes can be used to detect subtle changes in behaviour, while minimising any potential interference from a human observer’s perspective^32^. We detected a clear and rapid increase in the core body temperature as well as heart rate in all immune challenged mallards, which correlated well with the timepoints when the white blood cell composition and gene expression profiles were altered in the immune challenged ducks. In contrast, no significant differences in activity levels between the treatment groups were detected during the acute phase response.

The cost of an upregulated immune response is debated^40^, yet continuous long-term heart rate data are rarely reported in disease ecology studies. It is therefore still unknown, too, whether heart rate and associated energy expenditure increase significantly in birds during common infections, such as AIV^28^. Here we found that the heart rate of mallards became elevated during the acute phase response in all immunological treatments. As heart rate loggers may become more commonly used in ecological studies in the future^19^ we expect that we will soon gain additional insights into the magnitude, duration and function of elevated heart rates during infections in wild birds.

While a host’s physiological response to an infection is important, a behavioural response such as sickness behaviour is similarly essential and ubiquitous^17^. Unexpectedly, we did not find differences in activity patterns between the immune challenged and the control birds. Currently we do not know whether the lack of a difference is a result of the captive conditions of our experimental design, a subclinical course of the APR due to the lower pathogenicity of the stimulant compared to the true pathogen, or whether it also reflects the possibility that the movement patterns of wild mallards are not affected by some infections^23^. We will follow up on this question in a sequel study that will investigate movement patterns in free-ranging mallards.

While certain physiological and behavioural responses can be monitored remotely, others require a biological sample from the animal. Luckily, blood samples can often be obtained easily, non-destructively and repeatedly from animals upon capture. Here we inferred the immune status and health of mallards by observing changes in the number of leukocytes in the blood. We saw a change in white blood cell composition in all treatment groups throughout the experimental protocol, but to a much lower level in the control group. The change in the control group suggests that leukocyte composition changed either due to the injection of saline, and/or due to some stressful condition during the course of the experiment. Handling stress itself can alter the white blood cell composition in birds^41^. Nevertheless, the change in heterophil:lymphocyte (H:L) ratio was more pronounced in all treatment groups than in the control group (Fig. 2) and correlated well with the timeline of the acute phase reaction.

We deliberately focused both on duration and magnitude of the acute phase response in the mallards to provide guidelines on when to measure the response when using these pyrogens. The peak body temperature in mallards was already observed after 3–4.5 h post stimulation, similar to what was previously found in Pekin ducks (*Anas platyrhynchos domesticus*)^16,42^. Likewise, the differential gene expression and the leukocyte count analyses show that the timing of the fever response correlated well with the number of genes that were differentially expressed in the treatment groups (Fig. 1a and Supplementary Information S3) as well as the increase in H:L ratio (Fig. 2). In future studies, focus could thus be given to one or a few of the characteristics measured here.

Our study is a first step to understand the immunocompetence in truly wild animals. While our initial studies examined immune responses of a non-domestic species under controlled conditions, we are aware that subsequent studies will need to be done entirely in the wild. Our strategy here was to reduce the complexity of the environment and to induce an immune response using immunostimulants. These immunostimulants are non-infectious compounds that trigger an immune response, but do not make the animal an infectious carrier. They can therefore be used in field experiments to study the immune response without spreading infectious agents^37^. In our study we aimed to test if immunostimulants (poly I:C, LPS and inactivated *S. aureus*) activate pathogen-specific gene regulatory networks in mallards.

Several of the most highly differentially expressed genes in the poly I:C treatment (Fig. 3a) are interferon stimulated genes (ISGs) that are activated during viral infections in ducks, including viperin (*RSAD2*), *IFITM1* (Interferon induced transmembrane protein 1), *IFIT5* (Interferon induced protein with tricopeptide repeats 5) and *OASL* (2′–5′ oligoadenylate synthetase-like)^43^. Poly I:C also induced a rapid and sustained upregulation of the *IFI6* (IFN-α-inducible protein 6) in the ducks, an effector that blocks the replication of flaviviruses such as West Nile virus^44^. This indicates that poly I:C induced a typical antiviral response in the mallards. Several of the most upregulated genes in the LPS treatment group are involved in defence response, including *TREM2* (triggering receptor expressed on myeloid cells 2), *IL1R2* (Interleukin-1 receptor 2), *PTX3* (Pentraxin 3), *LYG2* (lysozyme G2) and *IL22RA2* (Interleukin 22 Receptor Subunit Alpha 2) (Fig. 3b). The most upregulated gene in the LPS treatment group (*PTX3*) was recently proposed as an important marker to monitor inflammatory conditions in poultry, as it is upregulated in response to bacterial and viral infections in chickens^45^. While the role of PTX3 is largely unknown in ducks, it was upregulated during early stage of egg drop syndrome virus infection in duck embryo fibroblast cells^46^. Interestingly, no significant upregulation of *PTX3* was detected in the mallards in response to poly I:C or inactivated *S. aureus* (Supplementary Datasets S1 and S3). While PTX3 is likely a good marker for LPS in mallards, its role in antiviral and antibacterial response in mallards thus needs to be further investigated. Another highly upregulated gene in the LPS treatment group (IL1R2), was recently proposed as a biomarker for differentiating gram-negative and gram-positive bacterial infections in mice, as this gene was expressed at a higher level in mice challenged with inactive gram-negative bacteria (*Escherichia coli*) than inactive gram-positive bacteria (*S. aureus*)^47^. In line with the results in mice, *IL1R2* was differentially expressed in the ducks treated with LPS (Supplementary Dataset S2) but not with the inactivated *S. aureus* (Supplementary Dataset S3). However, as few genes were highly up- or downregulated in the mallards treated with inactivated *S. aureus* (Supplementary Dataset S3) more research is required to determine if IL1R2 is also a good marker for differentiating gram-negative and gram-positive bacterial infections in mallards.

One of the top ten differentially expressed genes in the *S. aureus* treatment group (*LYG2*), was also differentially expressed in the LPS and the poly I:C treatment group (Supplementary Dataset S1–S2). In fact, this was one of the few genes that was differentially expressed in all treatment groups (Supplementary Dataset S1–S3). While the role of this gene in the duck immune response is unknown, *LYG2* is upregulated in response to bacterial^48^ as well as viral^49^ infections in chicken. It is thus plausible that this gene may be involved in the immune response to a broad range of pathogens in ducks as well.

Results from our gene ontology analyses show that the immunological stimulants induce pathogen-specific changes, which justify their use as surrogates to live pathogens in future manipulative studies. Several antiviral pathways were overrepresented in the poly I:C treatment groups, including the RIG-I/MDA5 signalling pathway (Fig. 4, Supplementary Dataset S5). RIG-I and MDA5 are pattern recognition receptors that recognise RNA viruses in the cytoplasm^50^, and activate a cascade of immune proteins which subsequently triggers the production of type I interferons^51^. The RIG-I/MDA5 signalling pathway is involved in the clearance of viruses with high relevance for mammals as well as birds^50,51^, and are upregulated in ducks infected with AIV^52,53^, Newcastle disease virus^54^, duck hepatitis virus^55^, and duck plague virus^56^. Considering that the mallard is a vector for viral diseases with major impact on human health and that several antiviral pathways were upregulated in the poly I:C treatment group (Fig. 4), this stimulant will be of particular interest for future ecological immunology studies in mallards. We also found that several pathways and biological processes related to immune function, inflammation and stress response were activated in the LPS treatment group (Fig. 4, Supplementary Information Figure S5), as has been seen in passerines^20,57^. Interestingly, although inactivated *S. aureus* (the Gram-positive bacterial mimic) induced an increase in body temperature and heart rate as well as a change in leukocyte composition, only a low number of differentially expressed genes (DEGs) were detected in mallards stimulated with this pyrogen. We think that we are only observing a part of the immune response usually triggered by live *S. aureus* infection, as live *S. aureus* activate the NGF $\upbeta$-TRKA signaling axis following stimulation of the NLRP3 inflammasome^58^. Therefore, we suggest that LPS, but not inactivated *S. aureus*, has a great potential for mimicking bacterial infections in ecological immunology studies in mallards.

Many of the genes that were differentially expressed in the mallards in our study are uncharacterised, and could not be identified using a similarity search against the other genomes used in our study. This demonstrates that there is still a lot to learn about the immune system in birds as well as some important model species. Further, the function of some genes that were differentially expressed in the mallards is unknown. One such example is the *B4GALNT4* (Beta-1,4-*N*-Acetyl-Galactosaminyltransferase 4) gene which was one of the most upregulated genes in the poly I:C treatment group (Fig. 3a, Supplementary Dataset S1, Supplementary Information Table S4). While mice deficient in B4GALNT3 (Beta-1,4-*N*-Acetyl-Galactosaminyltransferase 3), a paralog of B4GALNT4, have reduced protection against influenza virus^59^, the role of B4GALNT4 in the response to viral infections is unknown^60^. *B4GALNT4* is located next to the *IFITM3* (Interferon Induced Transmembrane Protein 3) gene, which is known to restrict influenza virus^61^. *IFITM3* is upregulated in mallards during influenza infection^61^, and was also one of the most upregulated genes in our poly I:C treated mallards (Supplementary Information Table S4). If further research supports our suggestion that B4GALNT4 is involved in the viral immune response in mallards, this gene is a good candidate for future functional studies with potential to improve our understanding of how mallards clear viral infections.

In many cases, the natural reservoir of EIDs show little to no signs of disease when being infected by the same pathogen that causes serious damage in other species^3^. Comparative transcriptomics and pathway analyses have great potentials for detecting subtle differences in the immune system that relate to specific differences in susceptibility or resistance to infections. If future studies move towards evaluating RNA-seq in the framework of pathways, then such differences will become more evident. By visualising the gene expression changes on these pathways for the mallard and creating an interactive webpage where the results can be evaluated (http://orn-files.iwww.mpg.de/dgeviz/) we provide means for future comparisons of the immune response in different species, including species with differences in severity of pathogenesis to AIV. When assessing gene expression in a pathway framework it is important to keep in mind that reference pathways are usually built on knowledge from model species such as human or mouse. The function of certain elements in the pathway used in this study might hence be different in the mallard, or even differ between the mallard and closely related species. One such example with relevance for this study is that birds lack the mammalian TLR6 and TLR9^62^. Other differences that will be of relevance for future comparative immunology studies in birds are that chicken, but not duck, lack the RIG-I and TRIF related adapter molecule (TRAM, also known as TICAM-2) proteins^53,63^. TRAM bridges the TLR4 and TRIF in the TLR3- and TLR4-mediated MyD88-independent signalling pathway, and is an important part of the TLR4 pathway^64^. As more genomic and transcriptomic studies are undertaken, a key next step will be the construction of species-specific immune regulatory networks for species with importance as hosts of EIDs.

In this study we used attached and implanted bio-loggers as well as blood-based assays to record several characteristics of the immune response simultaneously. While a combination of different measuring techniques is indispensable for obtaining a comprehensive picture of the immune response, each technique has inherent practical limitations. The heart-rate and body temperature bio-loggers used in this study require surgical implantation, which may not be feasible in certain species and field settings. Blood-based assays in turn may be available to more research groups, but baseline information may not be available for many reservoir species hampering the interpretation of results. The technology to use within a particular study thus has to be determined based on the ecology of the species of interest and the research question in mind. We expect that the continuous technological development (including smaller sensors, improved ability to transmit data, and novel attachment and recovery methods)^65^ combined with appropriate archiving and trans-disciplinary sharing of data (*e.g.* movebank.org) will facilitate the usage of bio-loggers for disease monitoring in the future.

In conclusion, we show that poly I:C and LPS induce a rapid and predictable acute phase response in mallards. We confirm that body temperature and heart rate increase during the acute phase response, and that this can be monitored remotely using on-animal bio-loggers. In combination with GPS data from tracking devices that can record movements of animals on a large scale^66^, we can now get closer to understanding the epidemiology of diseases such as AIV in mallards. By analysing the transcriptome after immune stimulation, we did not only gain novel insights into the molecular mechanisms behind this immune reaction but also showed that pathogen-specific immune pathways were upregulated in the blood during the acute phase response.

## Materials and methods

All methods are described in more detail in the Supplementary Information.

### Immune challenge

Forty-four first generation captive-bred mallards (*Anas platyrhynchos*) were included in the study. The mallards were housed in groups of three in outdoor aviaries at the Max Planck Institute for Ornithology (MPIO) in Radolfzell, Germany. The aviaries measured 3 m x 4 m × 2.5 m (w × l × h) and contained a water basin and a shelter with nesting material (Supplementary Information Text S9).We treated the mallards with one of three immunostimulants to mimic infections by different pathogens. The double-stranded RNA molecule polyinosinic:polycytidylic acid (poly I:C, 1 mg/kg) was used to mimic a viral infection, lipopolysaccharide (LPS, 100 µg/kg) was used to mimic a Gram-negative bacterial infection, and cell walls of heat-killed *Staphylococcus aureus* (approx. 2.5 × 10^10^ cell walls) were used to mimic a Gram-positive bacterial infection. These compounds are all used as common tools for scientific research on the immune response and have all been shown to induce an increase in body temperature in Pekin ducks (*Anas platyrhynchos domesticus*)^16^. As body temperature as well as heart rate can be elevated in birds during stress or handling^67^, the experiment was divided into two parts. The first part of the experiment (Experiment 1) allowed us to monitor changes in body temperature, heart-rate and movement patterns from the individuals without disturbance, while the second part of the experiment (Experiment 2) allowed us to collect blood samples that were used to study differential gene expression and white blood cell composition (Supplementary Information Figure S1).

In Experiment 1 we recorded changes in physiology and behaviour during the acute phase response using bio-loggers. For this purpose, we implanted heart rate and body temperature sensors (E-obs GmbH, Grünwald, Germany, www.e-obs.de) in the abdominal cavity of 12 individuals. Four weeks after surgery, we attached acceleration loggers (E-obs GmbH, Grünwald, Germany, www.e-obs.de) to the back of the same individuals using a customised backpack^68^. We divided the individuals into four groups of three individuals each, which then received one of the treatments. The individuals were left in the aviaries with minimal disturbances after stimulation to avoid changes in physiology and behaviour due to handling stress.

In Experiment 2 we repeated the treatments to collect blood samples for leukocyte counts and global gene expression analysis. An additional 32 mallards were included in the second stimulation event to ensure that enough samples were available for further analysis. The treatment was repeated after a minimum of two weeks to avoid potential short-term tolerance effects to the stimulants^16,69^. Once again, the individuals (n = 44) were divided into four groups (n = 11) and stimulated with one of the three treatments or the control. Blood samples were collected before stimulation and at a number of time points post stimulation (ps) (3 h, 6 h, 12 h, and 24 h).

For more detailed description see Supplementary Information (Text S9).

### Body temperature, heart rate, activity data and leukocyte composition

We used bio-loggers to observe changes in physiology and behaviour during the acute phase response. Briefly, we recorded body temperature, electrical activity of the heart, and acceleration data for three individuals per treatment. The data was downloaded remotely using an e-obs base station located outside the aviaries. We calculated the heart rate as beats per minute for every five-minute period from the electrocardiograms. We estimated the activity level of the mallards by calculating the mean of the variance of the acceleration measurements from each axis, following^70^. We fitted generalised additive mixed models (GAMMs) to the data for each physiological measurement, to investigate whether they differed between the treatments. We estimated the mean from the posterior distribution using a Bayesian framework for each measurement.

We performed a leukocyte differential count and calculated the heterophil:lymphocyte (H:L) ratio to get a better understanding of what changes occur in the leukocyte composition during the acute phase response in mallards. We prepared blood smears for five individuals per treatment and time point and determined the proportion of heterophils, lymphocytes, monocytes, eosinophils, and basophils using light microscopy. Staining and evaluation of blood films was performed by Pendl Lab, Switzerland. We fitted a multinomial model to estimate and compare the proportions of each leukocyte type in the different treatments and time points. The mean of the H:L ratio was estimated from the posterior distribution of the multinomial model.

We report the 95% Credible Intervals (CrI) using the 2.5% and 97.5% quantiles from the posterior distribution from each model. The mean of each measurement was considered different from the mean of the control group when the CrI of the treatment group did not include the estimated mean from the control group.

For more detailed description see Supplementary Information (Text S10, Table S8).

### Genome wide gene expression profiling

We monitored changes in the whole transcriptome in whole blood before and after the immune-stimulation for six individuals per treatment, using next generation RNA-sequencing to determine whether relevant immune pathways were upregulated in the respective treatments. We sequenced mRNA libraries on the Illumina HiSeq2500, and performed differential gene expression analysis using packages edgeR^71^ and limma^72^ as described in ^73^. Briefly, we computed empirical Bayes moderated t- and B-statistics, correcting for possible sex and individual differences using fixed and random factors, respectively, to identify genes that were differentially expressed due to treatment. Genes with an FDR adjusted *p* value < 0.05 were considered differentially expressed. We used Venn diagrams to explore whether the same genes were differentially expressed in the treatment groups and at different time points, and visualised the expression level for the differentially expressed genes (DEGs) using heatmaps. We conducted a gene ontology (GO) analysis in PANTHER^74^ to retrieve GO IDs^75^ for the DEGs and to find pathways that were overrepresented in each treatment group.

We visualised the gene expression data on seven immune related pathways (apla04620 Toll-like receptor signaling pathway, apla04621 NOD-like receptor signaling pathway, apla05132 Salmonella infection, apla05164 Influenza A, apla04623 Cytosolic DNA-sensing pathway, apla04672 Intestinal immune network for IgA production, apla04622 RIG-I-like receptor signaling pathway) from the KEGG database^34–36^ in the VANTED software^76^. All pathways were compiled into an interactive webpage (http://orn-files.iwww.mpg.de/dgeviz/). The web-based pathway visualizations contain hyperlinks to a description of each gene via the KEGG webpage, including the gene and protein sequence and links to the National Centre for Biotechnology Information (NCBI) and Ensemble.

For the poly I:C treatment we validated the RNA-seq results using real-time quantitative PCR (qPCR) (Supplementary Information Text S3), and thereby also provide a panel of target genes for specific future antiviral gene expression studies in mallards.

For more detailed description see Supplementary Information (Text S3, Table S9, Figure S10) and Supplementary Dataset S7.

### Ethics statement

The experiment was approved by the federal authorities of the German state of Baden-Württemberg (Regierungspräsidium Freiburg, approval no. AZ: 35-9185.81/G-15/130). Based on § 42 TierSchVersV (German legislative decree for the conduct of animal experiments) the approval of the authorities has to follow the votum of a commission for animal experiments. This commission is comparable to the ethical committees in other countries, but, according to German legislation, it is not appointed by the research institutes but by the state authorities. The study was carried out in compliance with the ARRIVE guidelines (https://arriveguidelines.org).

## Supplementary Information

Supplementary Data 1.

Supplementary Data 2.

Supplementary Data 3.

Supplementary Data 4.

Supplementary Data 5.

Supplementary Data 6.

Supplementary Data 7.

Supplementary Information.

## Data Availability

Raw Illumina sequences have been deposited at the NCBI’s Sequence Read Archive (SRA) database under the accession no. PRJNA728347.
