# Supplementary material for: Health monitoring in birds using bio-loggers and whole blood transcriptomics
Source: Sci Rep. 2021 May 24;11:10815. doi: 10.1038/s41598-021-90212-8 (PMC8144624; doi:10.1038/s41598-021-90212-8)
Supplement: Supplementary file 8 — Supplementary Information. [file 41598_2021_90212_MOESM8_ESM.pdf]

Supplementary Information for

Health monitoring in birds using bio-loggers and whole blood transcriptomics

Elinor Jax*, Inge Müller, Stefan Börno, Hanna Borlinghaus, Gustaw Eriksson, Evi Fricke, Bernd Timmermann, Helene Pendl, Wolfgang Fiedler, Karsten Klein, Falk Schreiber, Martin Wikelski, Katharine E. Magor, Robert H.S. Kraus

Elinor Jax*
Email: ejax@ab.mpg.de

This PDF file includes:

Supplementary Texts S1-S10
Supplementary Figures S1 to S10
Supplementary Tables S1 to S9
Captions for Supplementary Datasets S1 to S7
References for SI reference citations

Other supplementary materials for this manuscript include the following:

Supplementary Datasets S1 to S7

Contents

Supplementary information text 3
Supplementary Text S1 - Leukocyte composition 3
Supplementary Text S2 – Sequencing statistics 3
Supplementary Text S3 - Genome wide gene expression profiling 3
Supplementary Text S4 – Top DEGs 7
Supplementary Text S5 – Venn Diagrams 7
Supplementary Text S6 – Overrepresented pathways 7
Supplementary Text S7 – Biological processes 8
Supplementary Text S8 – Pathway visualizations 9
Supplementary Text S9 - Immune Challenge 9
Supplementary Text S10 - Body temperature, heart rate, activity and leukocyte composition 11
Supplementary information figures 14
Supplementary Figure S1 Timeline of the experiment 14
Supplementary Figure S2 Cumulative mean proportion of the different white blood cells 15
Supplementary Figure S3 Number of significantly differentially expressed genes (DEGs) 16
Supplementary Figure S4 Venn diagrams showing the overlap of significantly DEGs 17
Supplementary Figure S5 Heatmap illustrating overrepresented Biological Processes 18
Supplementary Figure S6 Toll-like receptor signaling pathway. 20
Supplementary Figure S7 Influenza A pathway 22
Supplementary Figure S8 Gene expression fold change real-time qPCR 23
Supplementary Figure S9 Correlation RNA-seq and real-time qPCR 24
Supplementary Figure S10 Multidimensional scale (MDS) plots 25
Supplementary information tables 26
Supplementary Table S1 Parameter estimates of the GAMM for body temperature 26
Supplementary Table S2 Parameter estimates of the GAMM for heart rate 26
Supplementary Table S3 Parameter estimates of the GAMM for activity level 27
Supplementary Table S4 The top significantly differentially expressed genes poly I:C 28
Supplementary Table S5 The top significantly differentially expressed genes LPS. 29
Supplementary Table S6 The top significantly differentially expressed genes *S. aureus* 30
Supplementary Table S7 NCBI BLAST top hits for the uncharacterised top DEGs 31
Supplementary Table S8 Staining protocol for blood films 32
Supplementary Table S9 Primers used for the real-time qPCR 33
Supplementary dataset legends 34
References 35

Supplementary information text

Supplementary Text S1 - Leukocyte composition

The composition of leukocytes changed in all treatment groups. The proportion of heterophils was increased, and the proportion of lymphocytes reduced at 3, 6, 12, and 24 hours post stimulation (hps) in the poly I:C treatment group (certainty >99%), at 6, 12 and 24 hps in the LPS treatment group (certainty >95%), and at 3 and 6 hps in the *S. aureus* treatment group (certainty >95%) than in the control group. In the poly I:C treatment group the mean proportion of monocytes was further lower than in the control group at 3 and 6 hps (certainty >99%). In the LPS treatment group the mean proportion of basophils was also lower in the stimulated group than in the control group at 6 and 12 hps (certainty >98%).

The estimated mean H:L ratio was higher in the stimulated groups than in the control group at 3, 6, 12, and 24 hps in the poly I:C treatment group (certainty >99%), at 3, 12 and 24 hps in the LPS group (certainty >95%), and at 3 and 6 hps in the *S. aureus* treatment group (certainty >96%).

Supplementary Text S2 – Sequencing statistics

The total number of sequencing reads per sample ranged from 28,210,306-84,361,960 with an average of 49,076,449 per sample. Of these, 86.75-91.69% successfully aligned to the reference genome. After filtering out genes that were not expressed at a biologically meaningful level in any treatment[1], 9822 genes remained for differential expression analysis. Of these, 7229 had an associated gene name in the reference annotation. An additional 1372 gene names were identified using the reciprocal best hits approach with BLAST (Dataset S4).

Supplementary Text S3 - Genome wide gene expression profiling

RNA isolation and RNA-sequencing

Tri Reagent BD (Sigma) supplemented with acetic acid (as described by the manufacturer) was added to 200 µL blood immediately after drawing. We mixed the samples by inverting and stored them at -80°C until further processing. The samples were later thawed on ice and processed according to the manufacturer's description. Briefly, we added the aqueous phase from the phase separation step in the Tri Reagent manual to a new tube, and processed the samples as described in step 4 (Load and Bind) and onwards in the peqGOLD Blood RNA Kit manual. We used the DNase I Digest Kit from peqGOLD to remove contaminating DNA, as described in the peqGOLD Blood RNA Kit manual. We assessed the quantity and quality of the RNA using the Qubit (Qubit® RNA BR Assay Kit) and Agilent 2100 Bioanalyzer system (Agilent RNA 6000 Nano Kit). We prepared mRNA libraries for the RNA (RIN value > 8.0) samples using the Illumina® TruSeq® RNA Sample Preparation Kit v2 and assessed the quality of the libraries using the Qubit system (Qubit® DNA HS Assay Kit, ThermoFisher Scientific), Agilent 2100 Bioanalyzer system (Agilent High Sensitivity DNA Assay Kit) and LightCycler® system (Lightcycler 480 II; Roche) (KAPA SYBR® FAST qPCR Kit Master Mix (2X) Universal). The libraries were sequenced on the Illumina HiSeq2500 (50bp, paired-end, 20mio sequence read pairs (fragments) per sample). The library preparation and sequencing were undertaken at the Max Planck Institute of Molecular Genetics (MPIMG) in Berlin, Germany. Initial processing of the data, such as removing adapters and filtering the reads by quality was further undertaken by MPIMG. The quality of the sequencing reads was assessed using FASTQC 0.11.4[2].

RNA-sequencing analyses

Alignment and quantification

The sequencing reads were aligned to the duck reference genome of Huang, et al. [3]; Anas_platyrhynchos.BGI_duck_1.0.dna.toplevel.fa and transcriptome (Anas_platyrhynchos.BGI_duck_1.0.84.gtf) which were both downloaded from Ensembl release 91 (ftp://ftp.ensembl.org/pub/release-86/fasta/anas_platyrhynchos). We used HISAT2 to index the reference genome and to align the short reads to the genome in paired-end mode[4]. We converted the output file to bam format, sorted the file and examined the alignment statistics using SAMTOOLS 1.3.1 (http://samtools.sourceforge.net). We then used the *SummarizeOverlaps* function from the *GenomicRanges* package[5], to quantify the number of reads that aligned to annotated genes (Anas_platyrhynchos.BGI_duck_1.0.84.gtf) from the bam files. This provided the raw count tables necessary for the downstream comparative gene expression analyses. We ran both HISAT2 and SAMTOOLS on a remote high-performance computing cluster "Universal Cluster" in bwHPC-C5 (http://www.bwhpc-c5.de/).

Identification of uncharacterised genes

The mallard reference genome contains annotation for 15,634 genes, of which roughly 6000 have a gene name associated to them. We used a reciprocal best hits (RBH) approach to identify orthologues to the uncharacterised genes. For this purpose, we extracted transcript sequences from the merged gtf file using the *gffread* command from the STRINGTIE package[6], and did a sequence similarity search against protein databases using the Basic Local Alignment Search Tool (BLAST)[7]. These transcripts were reciprocally searched using BLASTX and TBLASTN[7] against the protein databases for chicken *Gallus gallus*, turkey *Meleagris gallopavo,* zebra finch *Taeniopygia guttata*, and collared flycatcher *Ficedula albicollis*, as well as mouse *Mus musculus* and human *Homo sapiens* (all downloaded from Ensembl release 91). We identified RBHs using the python script reciprocal_blast_hits.py (https://scriptomika.wordpress.com/2014/01/28/extract-best-reciprocal-blast-matches/), e-values <= 10^{-3} in both directions were considered successful hits as suggested by [8]. We downloaded gene IDs for protein hits from Ensembl's (release 91) BioMart[9]. We replaced the gene IDs for the uncharacterised genes with the gene IDs from the RBHs, with decreasing priority for larger distance since species divergence (chicken, turkey, zebra finch, collared flycatcher, mouse and human; Dataset S4). To identify genes among the top ten differentially expressed genes that still remained uncharacterised after the RBH BLAST, we searched these transcripts against the NCBI database using nucleotide BLAST (Table S7).

Gene expression analysis

We performed differential gene expression analysis in R using the packages EDGER[10,11] and LIMMA[12], as described by Law, et al. [1]. Prior to the comparative analysis, we made multidimensional scaling (MDS) plots for the entire RNA-sequencing data set to visualise sample-to-sample distances solely on the basis of their gene expression profiles, using the plotMDS function in LIMMA[12]. In addition to treatment and individual, samples clustered according to sex in the multidimensional scaling (MDS) plots (*SI Appendix*, Figure S10).

We therefore compared gene expression between females and males for each treatment and time point, as well as for females and males separately, to investigate whether females and males responded differently to our treatments. While the differentially expressed genes differed slightly when doing the analyses for females and males separately, there were no significantly differentially expressed genes when comparing females and males. As our preliminary analyses did not detect a clear difference in the response between females and males, we included individuals from both sexes in the remaining analyses. The results from the female male comparison will be discussed in more detail in a separate research note. As part of the investigations comparing the female and male

immune response, we detected one individual with an upregulated immune response at the start of the stimulation. This female, belonging to the treatment group poly I:C, was excluded from further differential expression analysis.

As one female and one male per treatment group had heart rate and body temperature loggers implanted, we further tested whether this had an effect on the gene expression profiles in the mallards. In contrast to the other factors tested, no clear clustering was observed in the MDS plot for individuals with and without implant (*SI Appendix*, Figure S10). To confirm this result, we compared gene expression between all mallards with and without loggers before stimulation (mallards without loggers n=16 and mallards with loggers n=8). No significant differentially expressed genes were detected between the groups. As individuals with loggers did not appear as outliers in our preliminary analysis, and as they were evenly distributed in all treatment groups, they were all included in the gene expression analysis.

We computed empirical Bayes moderated t- and B-statistics with LIMMA[13], correcting for possible sex and individual differences using fixed and random factors, respectively, to identify genes that were differentially expressed due to treatment. The change in gene expression in each treatment group from time point zero to the respective time point ps was compared to the change in the control group between the same time points. Reported gene expression fold changes from the RNA-seq analysis were log2 transformed, and the p-values were Benjamini and Hochberg FDR adjusted p-values[14]. Genes with an FDR < 0.05 were considered differentially expressed. We used Venn diagrams to explore whether the same genes were differentially expressed in treatment groups and at different time points. We used the *coolmap* function within LIMMA[13] to visualise the expression level for the differentially expressed genes from each treatment in heatmaps.

Gene ontology analysis and enrichment test

We submitted lists containing the significantly differentially expressed genes for each treatment group and time point to PANTHER (Protein Analysis Through Evolutionary Relationship)[15], to retrieve gene ontology (GO) IDs for the genes of interest. We further performed the PANTHER overrepresentation test (released 05.12.2017) for each list to investigate whether certain GO terms were overrepresented in our list of differentially expressed genes. The GO term annotation was performed for the GO root categories *GO biological process complete* (GO Ontology database released 2017-12-27) and *Reactome pathways* (Reactome version 58 released 2016-12-07) using *Homo sapiens* (all genes in database) as reference lists. A Fisher's exact test with FDR multiple test correction was applied to each test.

Data mapping onto KEGG pathways

We downloaded all available KEGG pathways for *Anas platyrhynchos* related to “Immune system”, “Infectious diseases: Viral” and “Infectious diseases: Bacterial” from the Kyoto Encyclopedia of Genes and Genomes (KEGG) database[16-18] in the VANTED software[19]. We displayed the log2 fold changes from the differential gene expression analysis for each treatment on each pathway in VANTED[19]. In total, the expression data was visualised on seven pathways (apla04620 Toll-like receptor signaling pathway, apla04621 NOD-like receptor signaling pathway, apla05132 Salmonella infection, apla05164 Influenza A, apla04623 Cytosolic DNA-sensing pathway, apla04672 Intestinal immune network for IgA production, apla04622 RIG-I-like receptor signaling pathway[16-18]). The interactive pathway pictures were built using VANTED functionality similar to the protocol described in Junker, et al. [20]. For those cases where nodes or edges were missing after import to VANTED, they were manually added in accordance with the KEGG pathways[16-18] for *Anas platyrhynchos*. All pictures were compiled into an interactive webpage (http://orn-files.iwww.mpg.de/dgeviz/), through the export function in VANTED[19]. The web-based pathway visualizations contain hyperlinks to a description of each gene via

the KEGG webpage, including the gene and protein sequence and links to the National Centre for Biotechnology Information (NCBI) and Ensembl.

Gene expression of target genes

We measured gene expression of a number of genes using real-time qPCR to validate our RNA-seq results and to provide a panel of target genes for future gene expression studies in mallards. We chose genes that were upregulated in the poly I:C treatment, based on the RNA-seq results. We used three reference genes for low pathogenic avian influenza virus infection in mallards; *UBE2O* (Ubiquitin-conjugating Enzyme E2 O), *RPS13* (Ribosomal Protein S13) and *RPL4* (Ribosomal Protein L4)[21]. We measured differential gene expression for five immune genes of interest; *RSAD2* (Radical S-adenosyl Methionine Domain Containing 2), *IRF7* (Interferon Regulatory Factor 7), *TLR3* (Toll-Like Receptor 3), *TLR7* (Toll-Like Receptor 3) and *RIG-I* (Retinoic acid Inducible Gene I) in five females and four males from three time points (0h, 3h, and 6h) and one treatment (poly I:C).

We used published cDNA primer sequences for some genes RIG-I, RPS13 and RPL4; [21,22]. We designed the remaining primers, as primers were either not available for these genes, or did not meet the criteria set by the MIQE guidelines[23] in our setup. We designed cDNA primers using Integrated DNA Technologies (IDT) Real-Time qPCR Assay Entry, following the guidelines set by Prediger [24]. The sequences for the genes of interest were retrieved from Ensembl release 91 (Transcript ID: ENSAPLT00000004233, ENSAPLT00000006241, ENSAPLT00000009346, ENSAPLT00000013271, ENSAPLT00000015756). The primer sequences are shown in *SI Appendix* (Table S9). We tested the stability of the reference genes in our treatment and the efficiency of all the primer pairs.

The efficiency of each primer pair was evaluated by performing a six-step fourfold serial dilution of the primers, before mixing them with the PerfeCTa SYBR Green Fast Max™ and running a real-time qPCR on samples from one individual. The log-mean was calculated for each dilution triplet and the slope of each primer was determined. The efficiency value was calculated by 10(-1/slope) i.e. proving if each cycle doubles the amount of the selected gene product (efficiency=2)[25]. The efficiency values were converted to efficiency in % by which an efficiency of 2 is denoted as 100% efficiency. Only those genes with an efficiency of 80-100% were included in the study.

To determine if any other products than the selected gene product had been amplified, two assessment procedures were undertaken. First, a melt curve analysis was applied to all real-time qPCR runs to assess at what temperature the dsDNA of the qPCR product dissociated. For this purpose, the temperature was increased from 60-95°C[26]. Second, a gel electrophoresis was performed on the qPCR products from all primer pairs. This was implemented to make sure that the primers were specific for one gene which in turn created one band in the agarose gel. To confirm the presence of a single amplicon 10 µl PCR product was mixed with 2 µl Thermo Scientific™ 6x MassRuler DNA Loading Dye and added to a 1% agarose gel consisting of 1x TAE-buffer, agarose and 3 µl Roti®-Gelstain. All gels were run on 80V for 30 minutes in 1x TAE-buffer with 5 µl Thermo Scientific™ MassRuler Low Range DNA ladder 60.8 ng/µl as a mass ruler.

We tested the stability for four reference genes (*GAPDH*, *RPS13*, *UBE20* and *RPL4*) across the three time points (0h, 3 hps and 6 hps) in the poly I:C treatment group. Samples from two individuals, one male and one female, were run on real-time qPCR and Cq-values were compared across the time points. Comparison between the samples was done with calculations of standard deviation (SD) and relative standard deviation (RSD). The reference genes showing the least SD ≤1 Cq and RSD ≤5% where selected as reference genes for further experimental runs. This included the three reference genes reported in Table S9.

We synthesised cDNA from 1 µg total RNA using the qScript™ cDNA Synthesis Kit (Quanta Biosciences, Darmstadt, Germany). We executed the real-time qPCR runs with the CFX96™ Real-Time System and C1000™ Thermal Cycler (BioRad, Germany) using PerfeCTa SYBR Green Fast Mix™ (Quanta Biosciences, Darmstadt, Germany). We ran the real-time qPCR with triplets of each sample. Each run followed the same protocol of 1 cycle at 98°C for 2 minutes; 45 cycles at 98°C for 2 seconds and 60°C for 5 seconds; followed by a melting curve analysis, 60-95°C with +0,5°C/5 seconds. We calculated the quantification cycle (Cq) value of the reference genes for each individual at each time point. We determined the combined ΔCq for all reference genes and the individual ΔCq for each gene of interest by calculating the change from Cq of zero hours (before stimulation) to three and six hours after stimulation. We then estimated the relative quantitation of gene expression using the ΔΔCT method[27]. We measured gene expression in a total of nine individuals in the real-time qPCR (using a larger sample size than that used for the RNA-seq analysis. We compared the gene expression fold changes retrieved from the real-time qPCR and RNA-seq analysis for the five genes using the five individuals that were included in both the real-time qPCR as well as the RNA-seq analyses. For this purpose, we calculated the gene expression fold change for the five genes of interest for each individual from zero hours (before stimulation) to three and six hours after stimulation using the CPM values retrieved from the RNA-seq data for the poly I:C treatment group.

Supplementary Text S4 – Top DEGs

In the poly I:C treatment group several genes were part of the top DEGs at more than one time point post stimulation (ps), and many of the top DEGs were also significantly differentially expressed at other time points ps (SI Appendix, Table S4). In the LPS and *S. aureus* treatment groups few genes were part of the top DEGs at more than one time point ps, and few genes were significantly differentially expressed at several time points (SI Appendix, Table S5-S6). A list of all DEGs from each treatment and time point is shown in Datasets S1-S3.

Supplementary Text S5 – Venn Diagrams

We made Venn diagrams to visualise the overlap of differentially expressed genes between the treatments for each time point ps (Figure S4a) and between the time points for each treatment (Figure S4b). The overlap of differentially expressed genes was small to moderate, suggesting that the pyrogens induced different responses at the gene expression level (*SI Appendix*, Figure S4a). While the overlap of differentially expressed genes was large in the poly I:C treatment group at the different time points, the overlap was much smaller in the LPS treatment group (*SI Appendix*, Figure S4b). This shows that similar genes were differentially expressed in the poly I:C group over the first 12h of the acute phase response. In the LPS treatment group, however, different genes were activated at 3 and 6 hours post stimulation.

Supplementary Text S6 – Overrepresented pathways

In the poly I:C treatment group several signaling pathways related to toll-like receptors (TLRs) were overrepresented (Figure 4, Dataset S5). TLRs are a group of pattern recognition receptors (PRRs) that together recognise a wide range of PAMPs, and that make up an essential part of the innate immune system[28]. Once TLR receptors are activated, the TLR signaling pathway is initiated through intracytoplasmic toll/interleukin-1 receptor (TIR) domains, such as Myeloid Differentiation Primary Response 88 (MyD88), TIR Domain Containing Adaptor Protein (TIRAP), and TIR domain-containing adaptor inducing IFN-β (TRIF)[29]. The TLR pathways ultimately result in cytokine secretion and

inflammatory response. While TLRs are conserved across a wide range of taxa[30-32], there are certain taxon-specific differences in the TLR signaling pathway [32]. In birds, TLR1 recognises lipoproteins, TLR2 recognises peptidoglycan, TLR3 recognises double stranded RNA, TLR4 recognises LPS, TLR5 recognises flagellin, TLR7 recognises single stranded RNA, and TLR21, which is absent in mammals, recognises CpG motifs and chromosomal DNA[33,34]. There is further an avian and reptile specific toll-like receptor, TLR15[34], which has been shown to recognise a yeast-derived agonist[35]. In the poly I:C treatment group several signaling pathways related to TLR3 and TLR4 were overrepresented (Figure 4, Dataset S5). As TLR3 recognises double stranded RNA as well as poly I:C[36], we expected to see upregulation of this gene in the poly I:C treatment group. TLR3 is also upregulated in ducks during AIV, NDV, reovirus, and duck plague virus (DPV) infection[37-40]. The TLR4 pathway, which normally recognises LPS from Gram-negative bacteria was also upregulated in the poly I:C group (*SI Appendix*, Figure S6-S7). This is possibly because *TLR4* is activated by oxidised phospholipids (OxPLs) which are formed during inflammation[41], also during AIV infection[42]. *TLR7*, which recognises single stranded RNA, was downregulated in the mallards from the poly I:C treatment group when compared to the control group (*SI Appendix*, Figure S6-S7). *TLR7* is upregulated in ducks during viral infection with AIV, NDV and DHV[39,43,44]. We triggered the typical PRR pathways that are activated during viral infections in ducks (RIG-I, MDA5, TLR3, TLR4; Figure 4-5 and *SI Appendix* Figure S6-S7 - but not all, see TLR7, Figure S6-S7) with the poly I:C treatment and therefore propose that poly I:C can be used as a mimic for many relevant viral infections in mallards. Using the combination of genes that we optimised and used for the real-time qPCR, the expression of some of the genes in the overrepresented pathways can be evaluated in future studies.

Interestingly none of the TLR pathways were overrepresented in the bacterial treatments. We expected to see the activation of the TLR4 signaling pathway in the Gram-negative bacterial treatment, as LPS is a known agonist to TLR4[45]. The cytokines *CCL4*, Chemokine C-C motif Ligand 5 (*CCL5*) and *IL8* in the TLR pathway were upregulated in the LPS treated group (Dataset S6, http://orn-files.iwww.mpg.de/dgeviz/). Further, several biological processes within the TLR pathway were overrepresented. For example, the MAPK signaling pathway, which is involved in signal transduction, regulating intracellular events such as cell differentiation, division and death[46,47] was overrepresented. The I-κB kinase/NF-κB signaling pathway, which regulates the expression of numerous genes within the immune response[48,49] was also overrepresented. These pathways are, however, also part of other PRR pathways. From our results, we therefore cannot exclude that these biological processes and cytokines were upregulated through a different PRR than TLR4. We further expected to see the activation of the TLR2 signaling pathway in the Gram-positive bacterial treatment, as lipopeptide and peptidoglycan from Gram-positive bacteria are known agonists to TLR2[45,50]. This was, however, not the case, with NF-κB1A being the only gene in the TLR pathway that was differentially expressed in the *S. aureus* treatment when compared to the control group. Neither of the *TLR2* genes, which are duplicated in birds[51], were differentially expressed in this treatment group.

Supplementary Text S7 – Biological processes

The GO overrepresentation analysis showed significantly overrepresented biological processes in the poly I:C treatment group at 3 (n=373), 6 (n=210), and 12h (n=49) ps, and in the LPS treatment group 3 (n=416) and 6 hps (n=129) (Datasets S5-S6).

In the poly I:C treatment group a large number of biological processes related to T-cell activation, differentiation and selection were overrepresented (e.g. GO:0045060, GO:0043383, GO:0045589, GO:0033077, GO:0046631), as were processes related to interferon (GO:0032728, GO:0032647, GO:0060334) and lymphocyte activation (GO:0031294, GO:0002708, GO:0030098), and cytokine production (GO:0002718, GO:0001816, GO:0060759) (Dataset S5). Additionally, a number of

biological processes related to pathogen recognition receptor (PRR) pathways were overrepresented, such as regulation of RIG-I signaling pathway (GO:0039535), MyD88-independent toll-like receptor signaling pathway (GO:0002756), TRIF-dependent toll-like receptor signaling pathway (GO:0035666), and toll-like receptor signaling pathway (GO:0002224). There were further a large number of biological processes related to viral infection overrepresented in the poly I:C treatment group (e.g. GO:1903902, GO:0045069, GO:0016032, GO:0039531, GO:1903901). In the poly I:C treatment group two biological processes related to bacteria were also overrepresented (GO:0009617, GO:0002237) (Dataset S5).

Several of the overrepresented biological processes in the LPS treatment group have functions in inflammatory response and immune cell activation (Dataset S6). Further, some biological processes related to bacterial infections were overrepresented (e.g. GO:0009617, GO:0002237, GO:0071219, GO:0032496). Additional biological processes with functions related to localization of protein or RNA to chromosome, telomere or nucleus were overrepresented in the LPS group at 6 hps (e.g. GO:1904814, GO:0090685, GO:0090670, GO:1904871, GO:1904851, GO:0070202) (Dataset S6). No biological processes related to viruses were overrepresented in the LPS treatment group.

In the *S. aureus* treatment group, no biological processes were overrepresented.

The overrepresented biological processes with the highest enrichment score for each of these groups are shown in *SI Appendix*, Figure S5.

Supplementary Text S8 – Pathway visualizations

Toll-like receptor signaling pathway

The fold changes for the characterised genes from the differential gene expression analyses in the poly I:C treatment group are illustrated on the TLR signaling pathway from KEGG[16-18] (*SI Appendix*, Figure S6). Within the TLR signaling pathway the following genes were significantly upregulated at one or several of the time points; *AKT1* (AKT Serine/Threonine Kinase 1), *CCL4* (C-C Motif Chemokine Ligand 4, also called *MIP-1β*), *FADD*, *IFNAR2* (type I interferon receptor 2), *IL8*, *IRF7*, *MAP3K8* (Mitogen-Activated Protein Kinase Kinase Kinase 8), *MAPK11*, *MAPK12*, *NFKB1*, *Stat1* (Signal Transducer And Activator Of Transcription 1), *TLR3* (Toll-Like Receptor 3), and *TRAF3* (TNF Receptor Associated Factor 3) (Dataset S7). The following genes were significantly downregulated at one or several of the time points: *CHUK*, and *TLR7* (Toll-like receptor 7) (Dataset S7).

Influenza A

The fold changes for the characterised genes from the differential gene expression analyses in the poly I:C treatment group are illustrated on the Influenza pathway (*SI Appendix*, Figure S7). Within the Influenza pathway the following genes were significantly upregulated in the poly I:C treatment group at one or several of the time points; *ACTG1* (Actin Gamma 1), *ADAR* (Adenosine Deaminases Acting on RNA), *AKT1*, *Casp1* (Caspase 1), *HSPA2* (Heat Shock 70kD Protein 2), *MDA5*, *IFNAR2*, *IL8*, *IRF7*, *MAPK11*, MAPK12, *Mx2* (MX Dynamin Like GTPase 2), *NFKB1*, *RIG-I*, *RSAD2*, *SOCS3*, *Stat1*, *TLR3*, and *TRIM25* (Dataset S7). The following genes were significantly downregulated at one or several of the time points in the poly I:C treatment group; *PRKCA* (Protein Kinase C Alpha), and *TLR7* (Dataset S7).

Supplementary Text S9 - Immune Challenge

Birds and housing

A total of 44 mallards (*Anas platyrhynchos*) were included in the experiment. All individuals were born in captivity but were of the second generation of wild mallards. The majority of the birds were one year old, with a few exceptions of older birds that were distributed evenly across the treatment groups.

Initially the mallards had a body mass of 1105 ± 122.37 g (mean ± SD). The mallards were housed in the outdoor aviaries at the Max Planck Institute for Ornithology (MPIO) in Radolfzell, Germany. The aviaries consisted of separate compartments measuring 3 x 4 meters, with a height of 2.5 meters. Each compartment contained a water basin (1 x 1.5 meters, 0.3 meters deep) and a shelter with nesting material (wood shavings and straw). During the immune stimulation, the birds were kept in groups of three individuals in each compartment. The aviaries were grate fenced to one side such that all animals were kept under ambient light and temperature conditions and were provided with food (Lundi Wasservogelsticks, article no.100908, Lundi) and water *ad libitum*. Before the sampling events, birds were caught using a handheld net. All processes described in this experiment were performed between December 2016 and January 2017 and approved by the federal authorities of the German state of Baden-Württemberg (Regierungspräsidium Freiburg, approval no. AZ: 35-9185.81/G-15/130).

Treatments

We used three pyrogens in the study to mimic different pathogens. These compounds are used as common tools for scientific research on the immune response and have all been shown to induce an increase in body temperature in Pekin ducks (*Anas platyrhynchos domesticus*)[52]. The birds were intramuscularly stimulated with 1 mL of one of the three pyrogens, or with 1 mL phosphate-buffered saline serving as a control treatment. The first pyrogen, polyinosinic:polycytidylic acid (poly I:C), is used to mimic certain viral infections due to its similarity to double stranded RNA which is present in some viruses[53-55]. Each mallard received 1 mg/kg poly I:C (P9582, double-stranded homopolymer polyinosinic-polycytidylic acid potassium salt; Sigma-Aldrich). The second pyrogen, lipopolysaccharide (LPS), is the major component of the outer membrane of Gram-negative bacteria and is known to induce a strong innate humoral and cellular immune response in animals with sickness behaviour mediated by pro-inflammatory cytokines[56-59]. Mallards receiving the LPS treatment were injected with 100 µg/kg LPS (L2630, phenol extracted *Escherichia coli* 0111:B4; Sigma-Aldrich, St. Louis, MO). The third pyrogen, cell walls of heat-killed *Staphylococcus aureus,* was used to mimic a Gram-positive bacterium. This pyrogen induces fever in mice and ducks[60]. About 2.5×10^{10} cell walls from heat-killed *S. aureus* (S2014, Wood 46 strain; Sigma-Aldrich) were used for this treatment. We determined the concentration of cell walls using flow cytometry on a BD Accuri C6 flow cytometer (BD Biosciences) at the FlowKon facility at Konstanz University, Germany.

Experimental setup

As body temperature as well as heart rate can be elevated in birds during stress or handling[61-65], the experiment was divided into two parts. The first part of the experiment (Experiment 1) allowed us to monitor changes in body temperature, heart-rate and movement patterns from the individuals without disturbance, while the second part of the experiment (Experiment 2) allowed us to collect blood samples that were used to study differential gene expression and white blood cell composition.

To account for potential daily temporal patterns (circadian rhythms) in the measured characteristics [66,67], all individuals received the treatment between 09.00 a.m. and 09.30 a.m. Central European Time (CET) in the first experiment, and between 09.00 a.m. and 10.00 a.m. CET in the second experiment. The exact time of injection for each animal was noted and scored as timepoint zero for that particular animal. Animals from different treatment groups received the treatment concurrently. In the second experiment, blood samples were collected 3, 6, 12 and 24 hours ±15 minutes after the time point zero as calculated for each animal.

The experimental design is described in *SI Appendix* (Figure S1).

Experiment 1 - Recording of physiological measurements using bio-loggers

We recorded changes during the acute phase response using bio-loggers. For this purpose, we implanted heart rate and body temperature sensors (E-obs GmbH, Grünwald, Germany, www.e-

obs.de) in the thoraco-abdominal cavity of 12 individuals under sterile conditions and general anaesthesia (inhalational anaesthesia with isoflurane, 5% induction; 2.5 – 3.5 % maintenance). The heart rate and body temperature sensors weighed approximately 9 g, measured 27 x 22 x 17 mm, and recorded the heart rate using two silicone-coated electrodes that emerge from the device and were placed close to the heart. Preanesthetically, an opiod analgetic (Butorphanol, 1.5 mg / kg/ i.m.) and Ringer's solution (20 ml/ kg/ s.c.) was administered. The thoraco-abdominal cavity was opened with a midline incision following the *linea alba* and the sensor was placed caudoventrally to the liver. The surgical incision was closed with a two layer (muscle tissue and skin) absorbable suture (Monosyn 4/0, B.Braun, Melsungen, Germany). Post-surgery a non-steroidal anti-inflammatory analgetic (Meloxicam, 0.5 mg/ kg / p.o.) was administered for pain management. Four weeks after surgery, we divided the individuals into four groups of three individuals each. The individuals were housed together with the other individuals about to receive the same treatment, to avoid cross treatment effects. Acceleration loggers (E-Obs solar bird, www.e-obs.de) were attached to the back of the animals using a customised backpack[68], to record behavioural changes during the acute phase response. The acceleration loggers measured 50 x 20 x 12 mm and weighed 26g including the backpack. The individuals were left for a week to acclimatise to their new environment and loggers. The groups of three individuals then received one of the treatments (*SI Appendix,* Figure S1, Experiment 1). The individuals were left in the aviaries with minimal disturbances after stimulation to avoid changes in body temperatures and heart rate due to stress.

Experiment 2 - Blood immune assays

We repeated the treatments to collect blood samples for leukocyte counts and global gene expression analysis. The treatment was repeated after a minimum of two weeks to avoid potential short-term tolerance effects to the stimulants[52,69]. An additional 32 mallards were included in the second stimulation event to increase the total number of individuals for the gene expression analysis. Once again, the individuals (n=44) were divided into four groups (n=11) and stimulated with one of the three treatments or the control as detailed above. The 12 individuals from experiment one received the same treatment in experiment two as in the first round of the experiment. Blood samples (300 µL) were taken from the wing vein (*V. cutanea ulnaris*) or the caudal tibial vein (*V. metatarsea plantaris superficialis medialis*) before stimulation and at a number of time points post stimulation (ps) (3h, 6h, 12h, and 24h) (*SI Appendix,* Figure S1, Experiment 2). The blood was collected in K3 EDTA coated tubes (Sarstedt, Nümbrecht, Germany).

A total of 44 individuals were included in the second experiment (*SI Appendix,* Figure S1, Experiment 2). In this study we analysed RNA samples from 24 individuals using RNA-seq (six from each treatment group, three from each sex, of which one female and one male per treatment group were included in Experiment 1 and thus had a logger implanted and two females and two males were only included and Experiment 2 and did not have bio-loggers). We further analysed RNA samples from nine individuals using real-time qPCR (all individuals from the poly I:C treatment except for the female outlier and one sample that was lost during cDNA synthesis). As the real-time qPCR confirmed the results from the RNA-seq in the poly I:C treatment group, we did not repeat the real-time qPCR for the other two treatments.

Supplementary Text S10 - Body temperature, heart rate, activity data and leukocyte composition

We recorded the body temperature, electrical activity of the heart, and acceleration data for a period of 4.2 seconds every five minutes during experiment 1, for three individuals per treatment. The data recorded during the first stimulation (*SI Appendix*, Figure S1) was downloaded using an e-obs base

station located outside the aviaries. For the heart rate data an electrocardiogram was projected in the acceleration viewer software (Movebank, *www.movebank.org,* Max Planck Institute of Ornithology, Germany), and the heart beat detector Java program was used to detect and count the heart beats (Matthias C. Berger, Schäuffelhut Berger GmbH, www.schaeuffelhut-berger.de). Additionally, we manually checked each electrocardiogram, to ensure that the heart beats were correctly called by the program. We calculated the heart rate as beats per minute for every five-minute period. We recorded acceleration in three dimensions for each period of 4.2 seconds every five minutes, which resulted in a total of 40 values per axis per burst. We calculated the variance of the acceleration measurements for each axis per burst, and we used the mean of these three variance values as a measure of activity[70].

We fitted Generalised Additive Mixed Models (GAMMs) to the data for each physiological measurement, to investigate whether they changed due to the different treatments. As an increase in body temperature as well as heart rate was observed for all individuals (including the control group) at around 19 hps, we built all models using data from the time of stimulation until 18.5 hps. The means of the heart beats per minute and activity level were calculated for each hour before fitting the models to these measures. Due to the large variability of the amplitude of the heartbeat measure, we further log transformed the heart rate data before fitting the model.

Body temperature, heart rate, and activity level were included as response variables in the respective models. Treatment and time post stimulation were included as predictor variables for each model. "Individual" was included as a random factor in all models to correct for repeated measurements within the same individual. We ran the GAMMs in R[71] using the MGCV[72] and NLME[73] packages. To account for autocorrelation of sampling points across time, we used the *corAR1* function in NLME[74]. We estimated the mean for each measurement from the posterior distribution, using a Bayesian framework. We further report the 95% Credible Intervals (CrI) using the 2.5% and 97.5% quantiles [75] from the posterior distribution. The mean of each measurement was considered different from the mean of the control group when the CrI of the treatment group did not include the estimated mean from the control group.

We used stained blood films and light microscopy to look at changes in leukocyte composition during the acute phase response[76]. For this purpose, we prepared blood smears for five individuals per treatment and time point. We placed 5µL of whole blood in EDTA on a microscope slide and used the wedge smear technique to prepare the blood films[77]. Staining and evaluation of blood films was performed by Pendl Lab, Switzerland. Non-fixed, air-dried blood films were stained with a modified (*SI Appendix*, Table S8) Wright-Giemsa-staining protocol[78].

Microscopic evaluation was carried out on an Olympus BX 41 microscope with WHN 10x/22 oculars armed with a 40x objective (UPlanFL N 40x/0.75 Ph2/0.17/FN 26.5) and a 100x objective (UplanSApo/100x/1.40 oil/0.17/FN 26.5). For the differential count a minimum of 200 leukocytes was determined per blood smear, using the 40x objective. The total number of heterophils, lymphocytes, monocytes, eosinophils, and basophils out of these 200 leukocytes was determined and a relative differential count in percentages was calculated. The slides were counted in chronological order (not blinded). To decrease miscounting due to uneven distribution of different leukocyte subpopulations within the bloodfilm, counting was performed by viewing a line of consecutive fields perpendicular to the blood film direction from edge to edge in the area of the monolayer[79].

We fitted a multinomial model to estimate and compare the proportions of each leukocyte type in the different treatments and time points. In the model we included counts for each leukocyte type as the multinomial response variable, and treatment and time point ps as explanatory variables. We included "individual" as a random factor to account for repeated measurements and fitted the model using a Bayesian framework by Markov chain Monte Carlo (MCMC) simulations. For this

purpose we ran WinBUGS[80] in R using R2WinBUGS[81]. We ran a total of three Markov chains with 500,000 iterations with a burn in period of 50,000. The chains were thinned by 10 (nine out of ten observation were discarded), to reduce autocorrelation. We assessed the convergence visually, and using the R-hat value[82]. The mean proportion and CrI for each cell type and explanatory variable were estimated from the posterior distribution (135,000 simulations). The mean and CrI of the heterophil:lymphocyte ratio (H:L) was further estimated from the posterior distribution of the multinomial model. For pairwise comparison between different groups, we used the posterior probability of the hypothesis that one mean is larger than the other from the posterior distributions of both means. From this comparison, we report the certainty that the mean of one group is larger than the mean of the other group.

Supplementary information figures

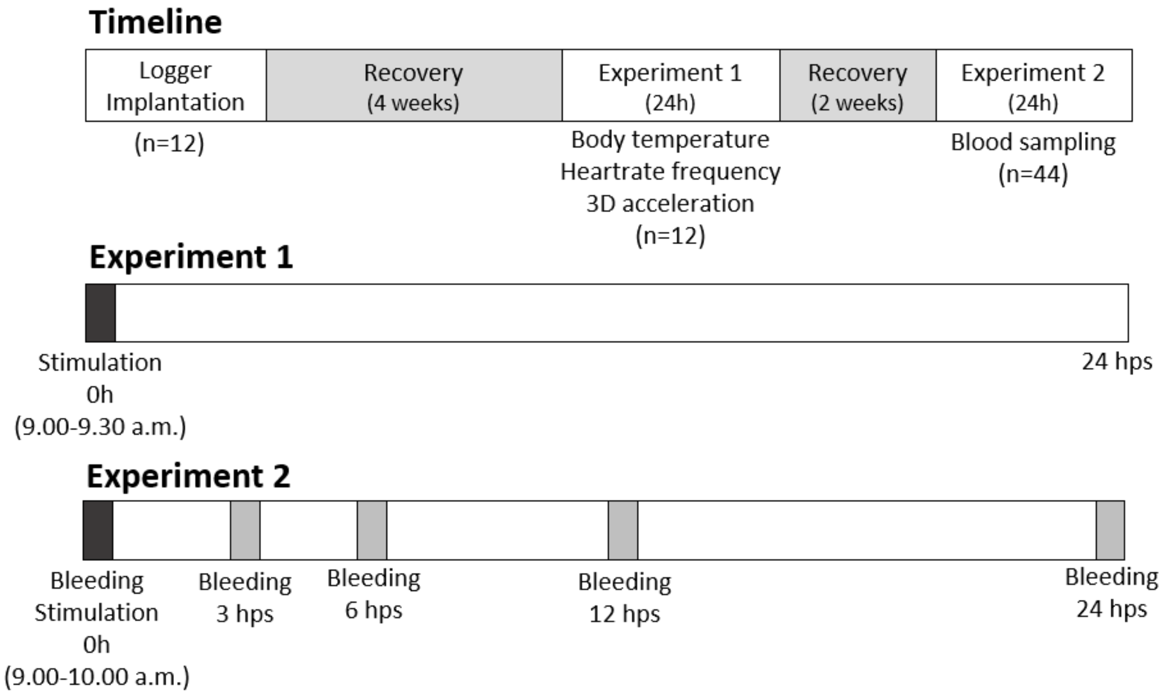

Supplementary Figure S1 Timeline of the experiment, showing when what events took place and the number of individuals that were included in the experiment; n = number of individuals, hps = hours post stimulation. For more details see Supplementary information Text S9.

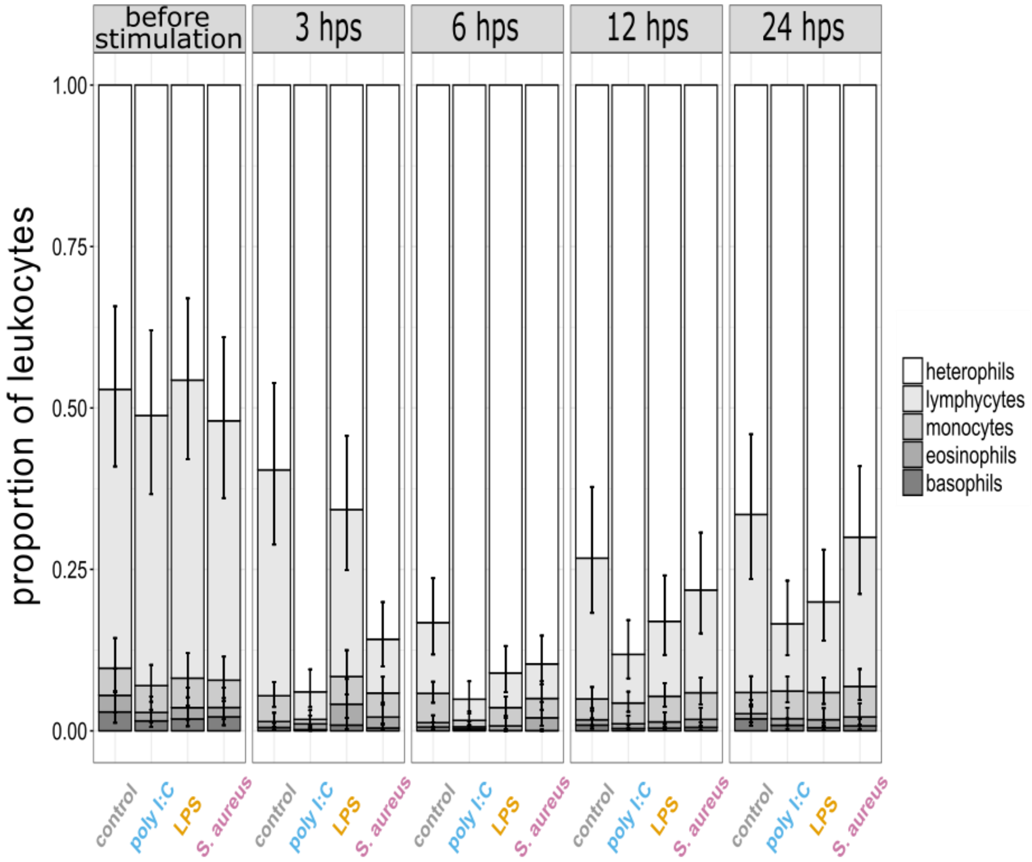

Supplementary Figure S2 Cumulative mean proportion of the different white blood cells and cumulative 95% Credible Interval as estimated from the posterior distribution of the multinomial model.

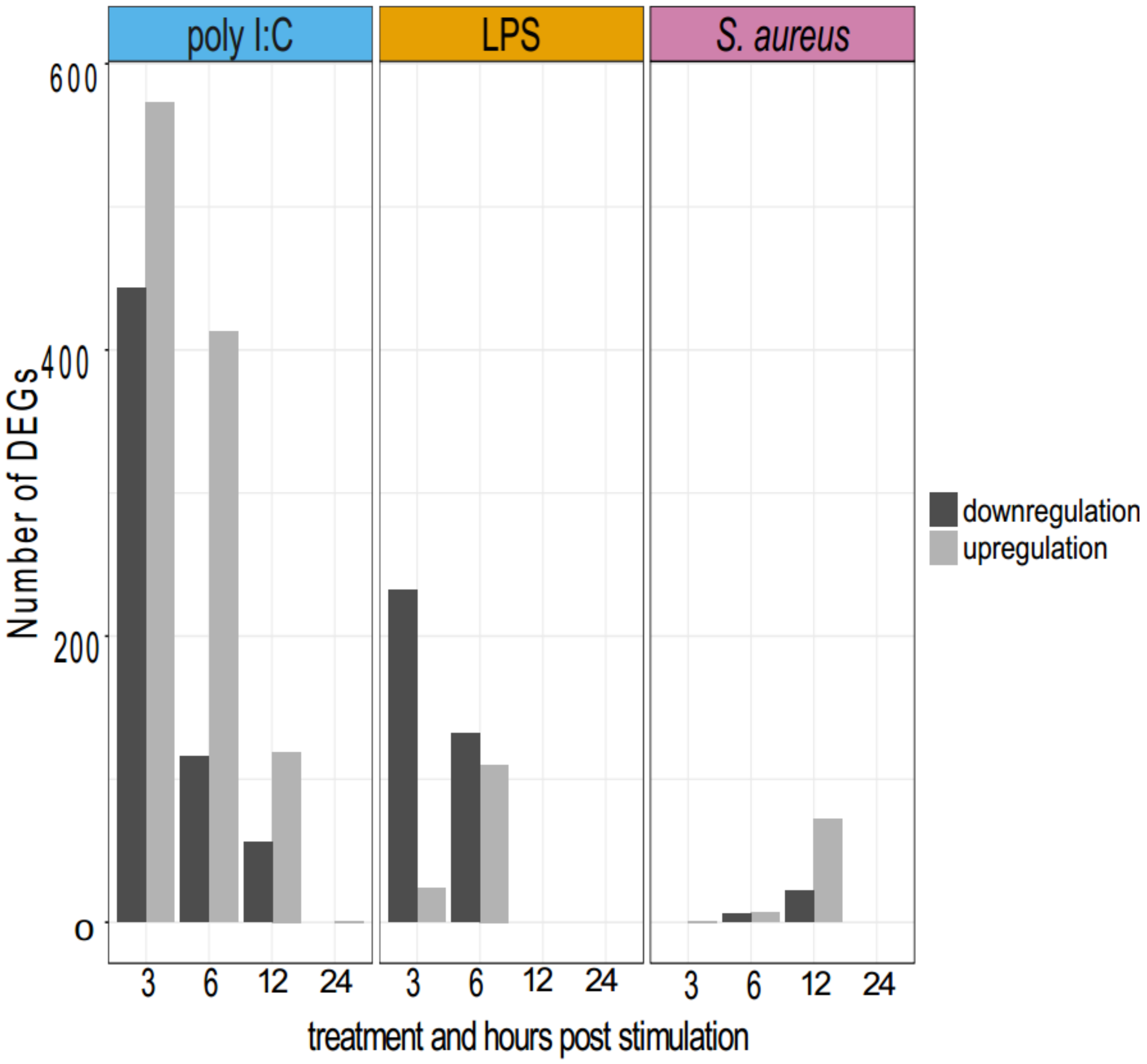

Supplementary Figure S3 Number of significantly differentially expressed genes (DEGs) for all treatment groups and time points (FDR adjusted p-value<0.05). Significantly differentially expressed genes that were upregulated when compared to the control group are shown in light grey, and genes that were downregulated when compared to the control group are shown in dark grey.

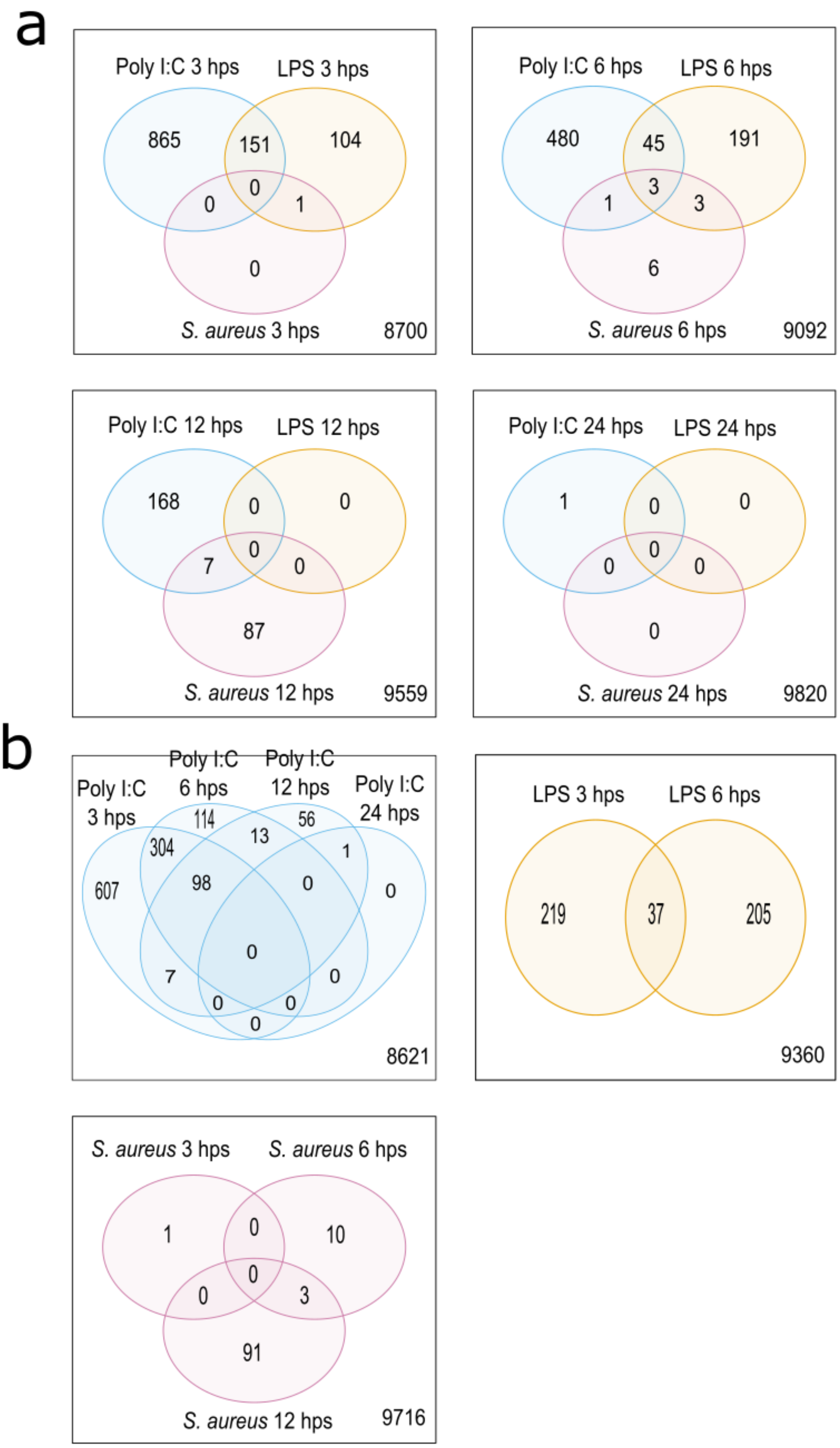

Supplementary Figure S4 Venn diagrams showing the overlap of significantly differentially expressed genes between a) each treatment for each time point and b) time points for each treatment

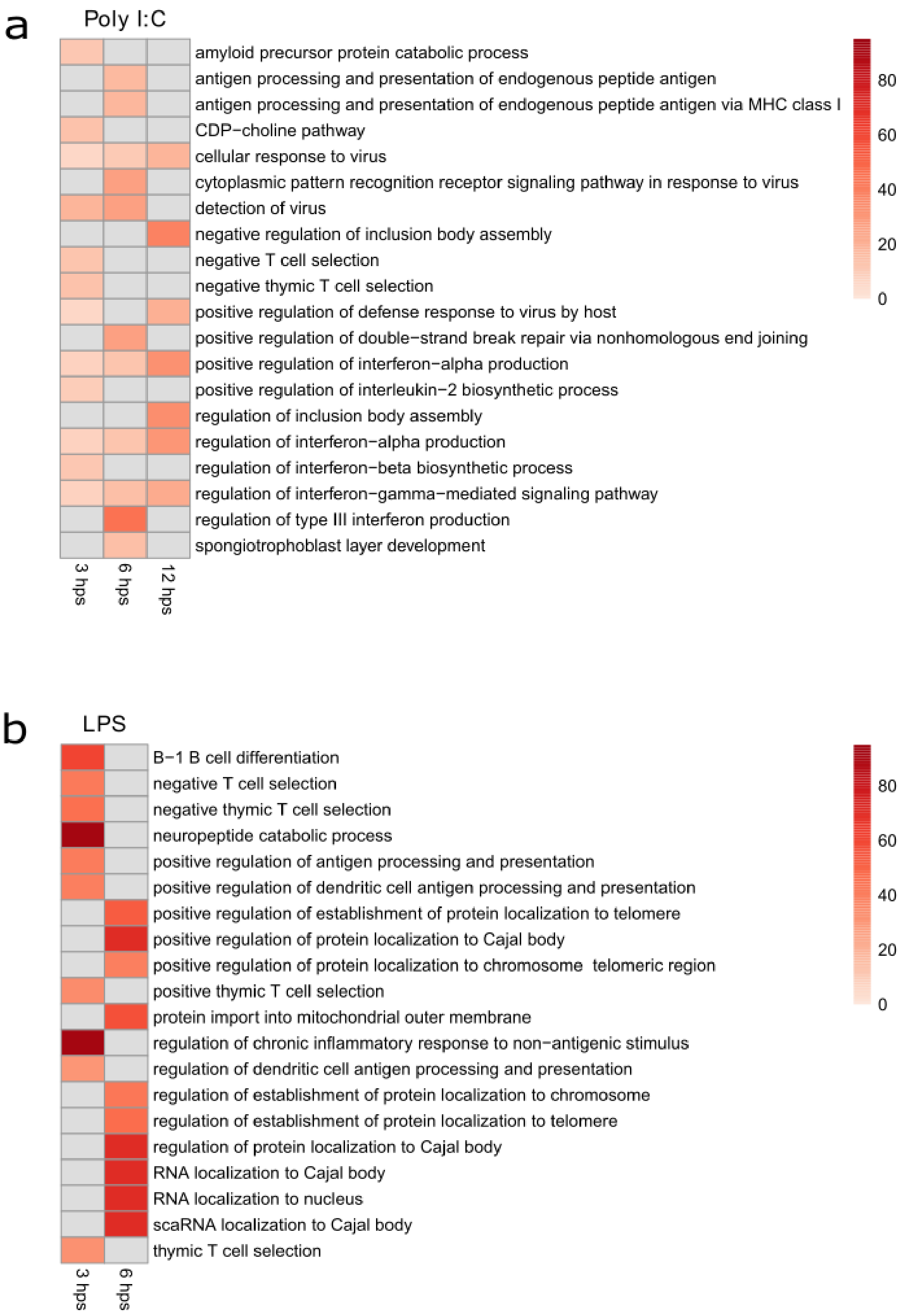

Supplementary Figure S5 Heatmap illustrating overrepresented Biological Processes with the highest fold enrichment score for the poly I:C and LPS treatment groups (FDR < 0.05)[15]. Pathways that were significantly overrepresented (FDR < 0.05) are shown in red colour, with faint red indicating lower fold enrichment score and dark red a higher fold enrichment score. Pathways that were not significantly overrepresented are shown in grey for that particular treatment group and time point. No Biological Processes were overrepresented in the *S. aureus* treatment group, nor 24 hps in the poly I:C

treatment group or 12/24 hps in the LPS treatment group. All overrepresented gene ontology terms are listed in Datasets S5-S6.

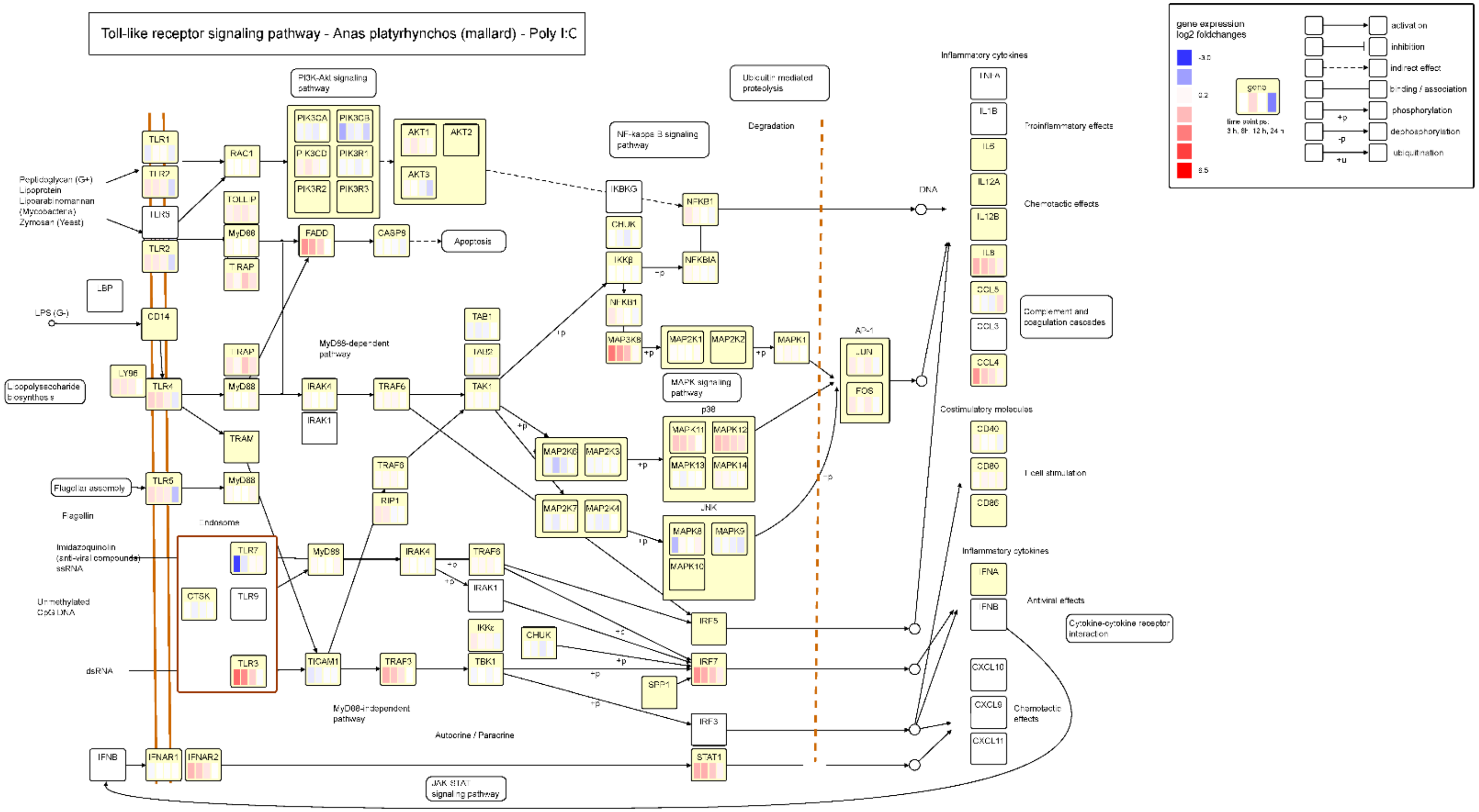

Supplementary Figure S6 Log2 fold changes of the expression level between the control and the poly I:C treatments mapped on the Toll-like receptor signaling pathway from the KEGG database[16-18]. Red indicates that the fold change was higher in the poly I:C treatment group than in the control group, while blue indicates that the fold change was lower in the poly I:C treatment group than in the control group. White indicates no change or a similar change in the poly I:C treatment group as in the control group. Paler shades of red and blue show a gradient of lower values in fold change. Each box represents one gene in the

pathway, and the columns within the box show the gene expression fold change for the four time points; 3, 6, 12 and 24 hps from the left to right. For more details seehttp://orn-files.iwww.mpg.de/dgeviz/TLR_PICButton.html.

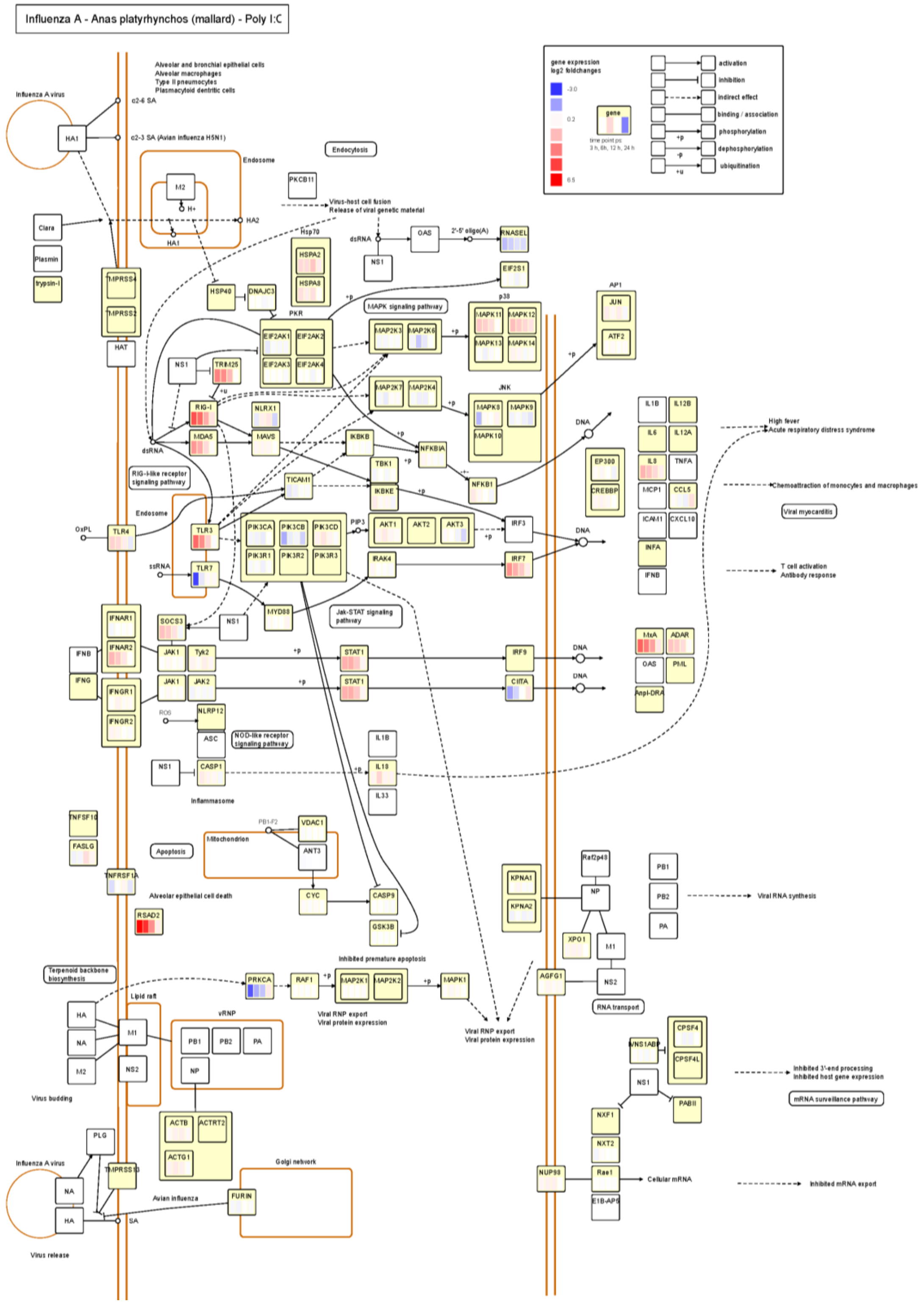

Supplementary Figure S7 Log2 fold changes of the expression level between the control and the poly I:C treatments mapped on the Influenza A pathway from the KEGG database[16-18]. Red indicates that the fold change was higher in the poly I:C treatment group than in the control group, while blue indicates that the fold change was lower in the poly I:C treatment group than in the control group. White indicates no change or a similar change in the poly I:C treatment group as in the control group. Paler shades of red and blue show a gradient of lower values in fold change. Each box represents one gene in the pathway, and the columns within the box show the gene expression fold change for the

four time points; 3, 6, 12 and 24 hps from the left to right. For more details see http://orn-files.iwww.mpg.de/dgeviz/AIV_PICButton.html.

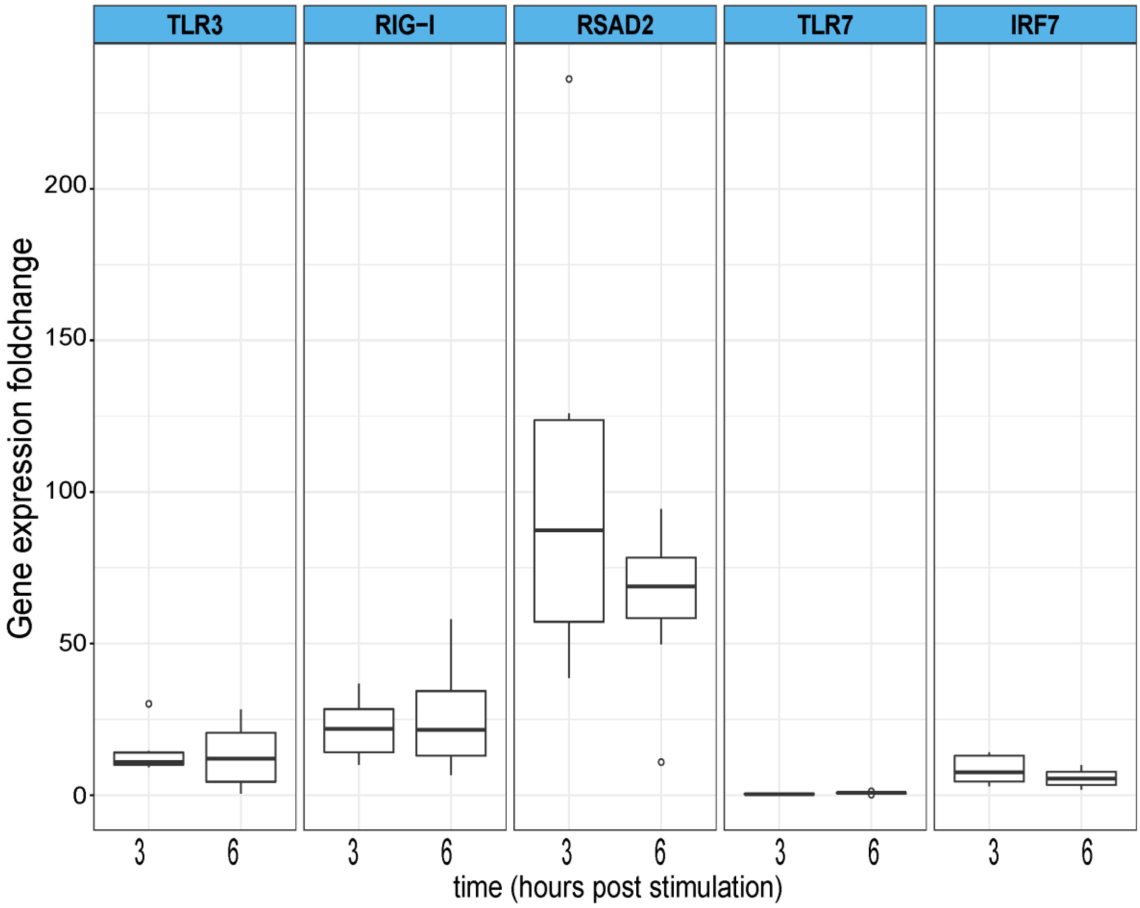

Supplementary Figure S8 Gene expression absolute fold change for TLR3, RIG-I, RSAD2, TLR7 and IRF7 as measured by real-time qPCR, calculated from nine individuals from the poly I:C treatment group. The box shows the median and the 25% and 75% quantile. The lower whisker shows the smallest observation greater than or equal to lower hinge -1.5 * Interquartile Range (IQR), while the upper whisker shows the largest observation less than or equal to upper hinge + 1.5 * IQR.

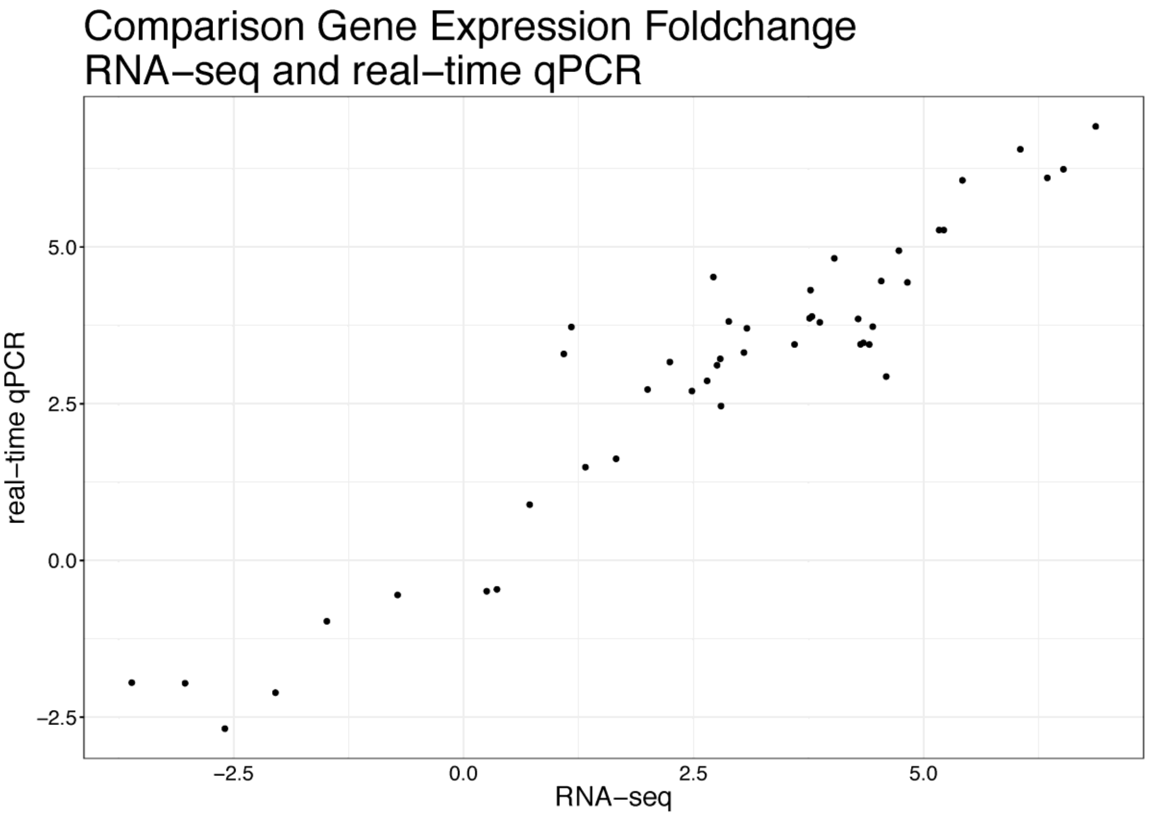

Supplementary Figure S9 Correlation of the gene expression log2 fold change in the poly I:C treatment measured using RNA-seq and real-time qPCR. Data for the five individuals included in both analysis and the five genes tested using both methods are plotted.

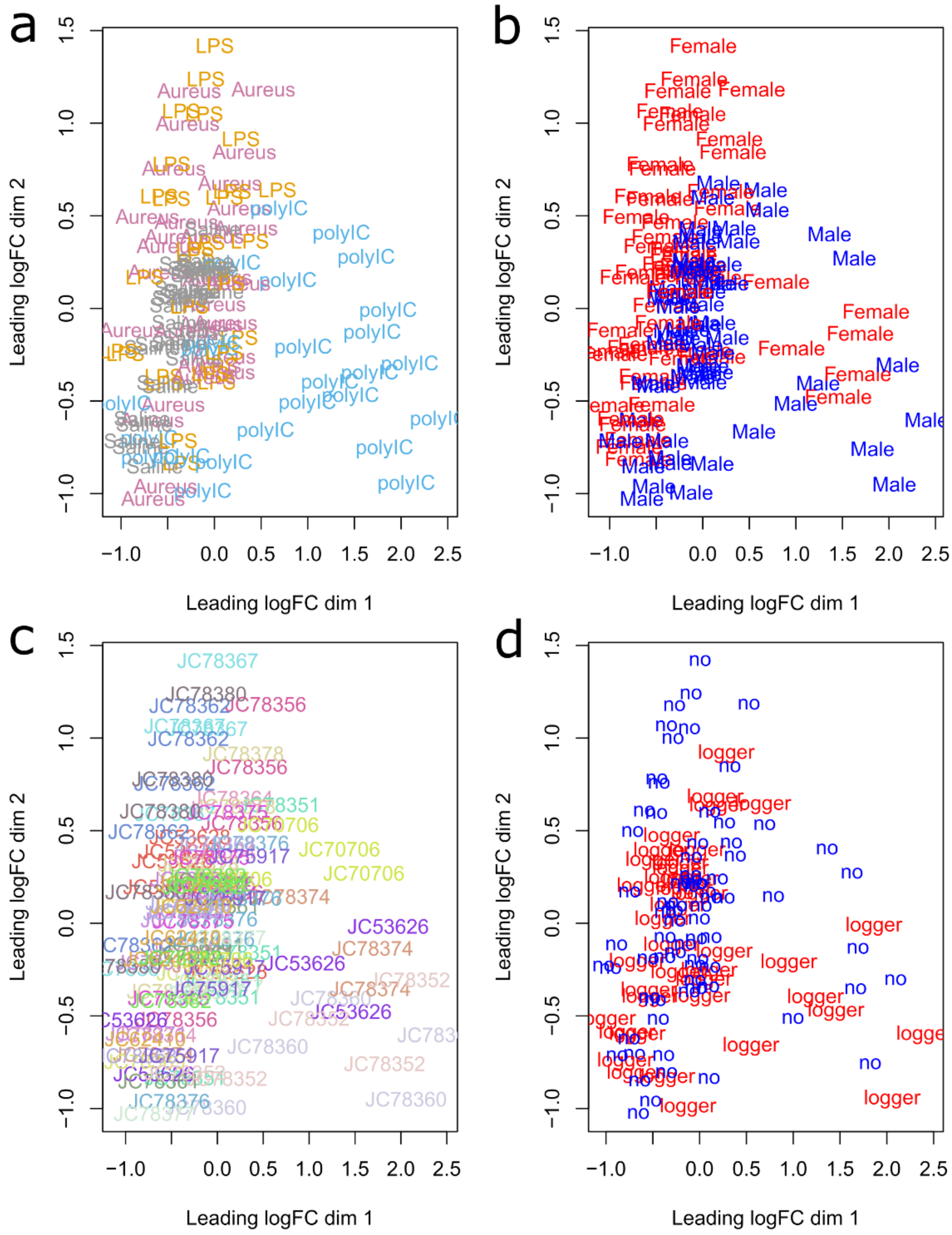

Supplementary Figure S10 Multidimensional scale (MDS) plots of log-CPM (counts per million) values for dimensions 1-2, with samples colored according to a) treatment, b) individual, c) sex, d) implant status. The distances on the plot correspond to the leading fold-change, which is the average (root-mean-square) log2-fold-change for the 500 genes most divergent between each pair of samples by default.

Supplementary information tables

Supplementary Table S1 Parameter estimates of the generalized additive mixed model (GAMM) with body temperature as dependent variable and treatment and hours post stimulation as predictors.

**A. Parametric coefficients**	**Estimate**	**Std. Error**	**t-value**	**p-value**
Intercept	39.7539	0.1969	201.892	< 2e-16
treatmentpolyic	0.8749	0.2785	3.142	0.001698
treatmentLPS	0.9617	0.2785	3.453	0.000562
treatmentAureus	1.5508	0.2785	5.569	2.82e-08
B. Smooth terms	edf	Ref.df	F-value	p-value
s(TIME):treatmensaline	8.510	8.510	22.70	<2e-16
s(TIME):treatmentpolyic	8.736	8.736	92.51	<2e-16
s(TIME):treatmentLPS	8.642	8.642	81.75	<2e-16
s(TIME):treatmentAureus	8.355	8.355	36.47	<2e-16

Supplementary Table S2 Parameter estimates of the generalized additive mixed model (GAMM) with heart rate as dependent variable and treatment and hours post stimulation as predictors

**A. Parametric coefficients**	**Estimate**	**Std. Error**	**t-value**	**p-value**
(Intercept)	4.75697	0.08247	57.685	<2e-16
treatmentpolyic	0.16942	0.11662	1.453	0.1464
treatmentLPS	0.14203	0.11662	1.218	0.2234
treatmentAureus	0.22232	0.11662	1.906	0.0567
B. Smooth terms	edf	Ref.df	F-value	p-value
s(TIME):treatmentsaline	1.000	1.000	0.611	0.434
s(TIME):treatmentpolyic	1.000	1.000	92.715	< 2e-16
s(TIME):treatmentLPS	3.466	3.466	45.859	< 2e-16
s(TIME):treatmentAureus	1.000	1.000	62.096	4.67e-15

Supplementary Table S3 Parameter estimates of the generalized additive mixed model (GAMM) with activity level as dependent variable and treatment and time post stimulation as predictors

**A. Parametric coefficients**	**Estimate**	**Std. Error**	**t-value**	**p-value**
(Intercept)	5.6736	0.2131	26.630	<2e-16
treatmentpolyic	0.4984	0.3013	1.654	0.0996
treatmentLPS	0.0754	0.3013	0.250	0.8027
treatmentAureus	-0.3538	0.3013	-1.174	0.2417
B. Smooth terms	edf	Ref.df	F-value	p-value
s(hour):treatmentsaline	1.000	1.000	0.960	0.32823
s(hour):treatmentpolyic	5.932	5.932	3.803	0.00152
s(hour):treatmentLPS	3.286	3.286	1.876	0.08605
s(hour):treatmentAureus	2.691	2.691	5.125	0.01542

Supplementary Table S4 The top significantly differentially expressed genes with the highest gene expression log2 fold change for each time point ps (post stimulation) in the poly I:C treatment group. The time points when each gene was significantly differentially expressed (adjusted p-value < 0.05) are shown in bold. For genes that could not be assigned a gene name from the duck genome but a reciprocal best hit (RBH) was found, the RBH is shown. For genes that could neither be assigned a gene name from the duck genome nor the RBH, the best hit from a NCBI BLAST is shown. All DEGs from the poly I:C treatment group are listed in Dataset S1.

**Duck ID**	**Gene**	**Orthologues gene**	**Mode of identification**	**Log2 Fold Change**			
				3 hps	6 hps	12 hps	24 hps
**ENSAPLG00000011142**	*IFI6*	ENSGALP00000022057.2	RBH	**7.43**	**8.17**	**7.36**	2.86
**ENSAPLG00000002006**				**7.21**	**5.01**	4.08	1.16
**ENSAPLG00000006016**	*RSAD2*			**6.48**	**5.29**	2.86	0.45
**ENSAPLG00000001610**	*IFIT1BL1 (IFIT5)*	ENSMUSP00000132781.1	RBH	**6.31**	**5.02**	2.82	0.41
**ENSAPLG00000011748**	*BCL2L15*			**6.3**	**4.26**	2.62	2.07
**ENSAPLG00000001534**	*PML*	101796105	BLAST NCBI	**6.19**	**5.22**	**3.75**	1.4
**ENSAPLG00000002170**	*CNP*			**5.94**	**4.68**	2.28	0.72
**ENSAPLG00000014996**	*IFITM1**	ENSGALP00000006737.4	RBH	**5.81**	**6.64**	**5.62**	2.66
**ENSAPLG00000015414**	*OASL*	101800895	BLAST NCBI	**5.68**	**4.96**	**3.37**	0.52
**ENSAPLG00000002196**	*OAS3*	ENSMUSP00000035588.8	RBH	**5.5**	**5.01**	**2.96**	0.26
**ENSAPLG00000011972**	*PLAC8*	ENSMGAP00000008232.1	RBH	**4.94**	**5.4**	**4.66**	1.87
**ENSAPLG00000005382**	*EPSTI1*			**4.85**	**4.8**	**3.31**	0.66
**ENSAPLG00000011216**	*HYDIN*	ENSGALP00000047727.1	RBH	**2.56**	**3.86**	**5.16**	1.42
**ENSAPLG00000014674**	*B4GALNT4*			**3.73**	**4.41**	**3.96**	2.02
**ENSAPLG00000016019**	*LAO1*	ENSGALP00000000108.5	RBH	**4.94**	**3.9**	**3.18**	0.36
**ENSAPLG00000014232**	*CYGNIN*	110354596	BLAST NCBI	**4.7**	**3.36**	**2.97**	0.12
**ENSAPLG00000001901**	*ADI1*			-0.04	0.19	**0.43**	**0.65**
**ENSAPLG00000005996**	*CMPK2*	ENSGALP00000042236.1	RBH	**5.19**	**4.11**	2.04	0.01
**ENSAPLG00000012605**	*TGM4*			**3.99**	**4.48**	1.95	0.48
**ENSAPLG00000003767**	*SDC4*			**4.84**	**4.05**	**2.72**	0.70

RBH = Reciprocal Best Hit
* Gene name changed from IFITM3 to IFITM1, following the suggested nomenclature in [83]

Supplementary Table S5 The top significantly differentially expressed genes with the highest gene expression log2 fold change for each time point ps (post stimulation) in the LPS treatment group. The timepoints when each gene was significantly differentially expressed (adjusted p-value < 0.05) are shown in bold. For genes that could not be assigned a gene name from the duck genome but a reciprocal best hit (RBH) was found, the RBH is shown. For genes that could neither be assigned a gene name from the duck genome nor the RBH, the best hit from a NCBI BLAST is shown. All DEGs from the LPS treatment group are listed in Dataset S2.

**Duck ID**	**Gene**	**Orthologues gene ID**	**Mode of identification**	**Log2 fold change**			
				3 hps	6 hps	12 hps	24 hps
**ENSAPLG00000004626**	*PTX3*			**8.25**	3.05	1.02	0.43
**ENSAPLG00000000744**	*MAS*	101797500	BLAST NCBI	**5.54**	2.85	1.45	0.91
**ENSAPLG00000004200**	*IL1R2*			**4.9**	3.33	1.67	1.42
**ENSAPLG00000010563**	*NETO2*			**3.55**	1.77	0.57	-0.06
**ENSAPLG00000000741**	*mas-related G-protein coupled receptor member H*	101795736	BLAST NCBI	**3.37**	3.11	1.2	0.67
**ENSAPLG00000007119**	*CD101*	101800493	BLAST NCBI	**-3.32**	-1.48	-2.14	-1.79
**ENSAPLG00000008731**	*TREM2*	ENSGALP00000039043.1	RBH	**-3.38**	-1.11	-0.44	-0.21
**ENSAPLG00000014182**	*HTRA1*			**-3.44**	-1.55	-1.17	-0.54
**ENSAPLG00000016361**	*DNASE2B*			**-3.45**	-0.45	-0.61	-0.76
**ENSAPLG00000002995**	*C1QB*			**-4.45**	-0.93	-0.7	-0.22
**ENSAPLG00000015164**	*Lyg2*	ENSGALP00000027012.2	RBH	0.63	**3.35**	0.97	0.23
**ENSAPLG00000001211**	*IL22RA2*			1.99	**3.33**	1.66	0.79
**ENSAPLG00000005304**	*SAA3*	ENSGALP00000010113.4	RBH	**1.79**	**2.98**	1.39	0.53
**ENSAPLG00000003584**	*CD1D*	101796317	BLAST NCBI	1.24	**2.96**	1.97	0.72
**ENSAPLG00000003674**	*CYGB*			1.93	**2.83**	0.68	-0.44
**ENSAPLG00000013942**	*SLC4A8*			2.72	**2.78**	0.89	1.25
**ENSAPLG00000016446**	*PLD4*			-1.64	**-2.73**	0.12	0.2
**ENSAPLG00000008309**	*NDST4*			**-2.49**	**-2.74**	-0.85	0.47
**ENSAPLG00000006283**	*BCL11B*			**-1.83**	**-2.79**	-1.21	-0.44
**ENSAPLG00000015598**	*IL9*	101800880	BLAST NCBI	-1.45	**-3.33**	-1.22	-0.08

Supplementary Table S6 The top significantly differentially expressed genes with the highest gene expression log2 fold change for each time point ps (post stimulation) in the *S. aureus* treatment group. The timepoints when each gene was significantly differentially expressed (adjusted p-value < 0.05) are shown in bold. For genes that could not be assigned a gene name from the duck genome but a reciprocal best hit (RBH) was found, the RBH is shown. For genes that could neither be assigned a gene name from the duck genome nor the RBH, the best hit from a NCBI BLAST is shown. All DEGs from the *S. aureus* treatment group are listed in Dataset S3.

Duck ID	Gene	Orthologues gene	Mode of identification	Log2 fold change			
				3 hps	6 hps	12 hps	24 hps
**ENSAPLG00000002420**	*SLC25A25*			**0.68**	0.32	0.21	0.09
**ENSAPLG00000015164**	*LYG2*	ENSGALP00000027012.2	RBH	-0.03	**2.36**	1.74	-0.27
**ENSAPLG00000010198**	*TGM3*	ENSMGAP00000004952.2	RBH	1.18	**1.41**	1.08	0.43
**ENSAPLG00000004736**	*SKA1*			0.2	**1.15**	0.98	0.29
**ENSAPLG00000001221**	*SLC5A3*			0.2	0.2	**0.76**	0.49
**ENSAPLG00000016180**	*B4GALT1*			0.41	0.41	**0.55**	0.29
**ENSAPLG00000008495**	*STK38L*			-0.58	-0.58	**-0.84**	-0.26
**ENSAPLG00000014306**	*ATIC*			-0.73	**-1.05**	-0.42	-0.38
**ENSAPLG00000008858**	*MORC4*			-0.58	-0.58	**-1.06**	**-0.89**
**ENSAPLG00000007308**	*IL7R*			-1.32	**-1.86**	-0.21	0.32
**ENSAPLG00000010562**	*CD3G*	ENSGALP00000011984.3	RBH	-1.31	**-1.87**	0.1	0.26
**ENSAPLG00000005998**	*ALMS1*	ENSGALP00000059752.1	RBH	-2.03	**-2.73**	**-2.01**	-1.64
**ENSAPLG00000014111**	*EPM2A*			0.27	0.27	0.71	**1.04**
**ENSAPLG00000005690**	*GIPC2*			0.58	0.58	0.8	**0.94**
**ENSAPLG00000008636**	*MLLT6*			-0.58	-0.58	-0.63	**-0.9**
**ENSAPLG00000007357**	*NUF2*	ENSMGAP00000003305.1	RBH	-0.51	-0.55	**-0.95**	-0.52
**ENSAPLG00000011102**	*NT5E*	101792766	BLAST NCBI	0.36	-0.29	**-1.15**	-0.26
**ENSAPLG00000007230**	*ALAD*			-0.61	-0.61	-0.97	**-1.27**
**ENSAPLG00000015291**	*ENTPD2*	101790941	BLAST NCBI	-1.39	-1.34	**-1.54**	-0.37
**ENSAPLG00000009934**	*MTR*			-0.98	-1.16	**-1.62**	-0.33

Supplementary Table S7 NCBI BLAST top hits for the uncharacterised top DEGs

Treatment	**Query sequence**		**Top hit from NCBI BLAST**						
	**Ensembl Gene ID**	**Ensembl Transcript ID**	**Gene name**	**Max Score**	**Total Score**	**Query cover**	**E-value**	**Per. Ident**	**GeneID NCBI**
Poly I:C	ENSAPLG00000001534	ENSAPLT00000001523	*Promyelocytic Leukemia (PML)*	1118	1118	99%	0	100%	101796105
	ENSAPLG00000015414	ENSAPLT00000016054	*2'-5'-oligoadenylate synthase-like protein 2 (OASL)*	747	868	100%	0	97.51%	101800895
	ENSAPLG00000014232	ENSAPLT00000014821	*cygnin*	344	344	100%	6.00 E-91	99.47%	110354596
LPS	ENSAPLG00000000744	ENSAPLT00000000720	*proto-oncogene Mas*	1663	1663	100%	0	99.67%	101797500
	ENSAPLG00000000741	ENSAPLT00000000716	*mas-related G-protein coupled receptor member H*	1722	1722	99%	0	99.47%	101795736
	ENSAPLG00000007119	ENSAPLT00000007379	*immunoglobulin superfamily member 2 (also called CD101)*	2809	2809	100%	0	99.10%	101800493
	ENSAPLG00000003584	ENSAPLT00000003656	*antigen-presenting glycoprotein CD1d*	664	820	94%	0	99.45%	101796317
	ENSAPLG00000015598	ENSAPLT00000016249	*interleukin-9 receptor-like (IL9)*	1040	1040	100%	0	98.33%	101800880
S. aureus	ENSAPLG00000011102	ENSAPLT00000011549	*cytosolic 5'-nucleotidase 1A*	1574	1574	100%	0	98.76%	101792766
	ENSAPLG00000015291	ENSAPLT00000015945	*ectonucleoside triphosphate diphosphohydrolase 2*	2518	2518	100%	0	97.27%	101790941

Supplementary Table S8 Staining protocol for blood films

**Colorant**	**Staining protocol**
3 g Wright-powder[1]<br>0.3 g Giemsa-powder[2]<br>5 ml glycerine<br>1000 ml absolute methanol (aceton free)[3]<br>filter and store in a dark vial<br>stable for several weeks	use air-dried native blood films<br>flood blood film 3 min with colorant<br>add equal amount of buffer pH 6,8[4]<br>mix gently by blowing with a pipette or a straw until metallic green sheen appears on the surface<br>allow to stand for 6 min<br>rinse and flood with buffer for 1 min<br>wash copiously with buffer<br>wipe the back of the blood film to remove excess stain<br>prop in rack until dry or use hair dryer<br>mount with Entellan®[5] and cover glass
[1]Merck® No 1.09278.0025<br>[2]Merck® No 1.09203.0025<br>[3]Merck® No 1.06009.1000<br>[4]Merck® No 1.11374.0100<br>[5] z. B. Entellan® Neu, Merck® No 1.07961.0100	

Supplementary Table S9 Primers used for the real-time qPCR

Gene Symbol	Gene Name		Primer sequence (5' - 3')	Reference
**Reference Genes**				
RPL4	Ribosomal protein L4	F	CCTGGGCCTTAGCTGTAACC	[21]
		R	AAGCTGAACCCATACGCCAA	
RPS13	Ribosomal protein S13	F	AAGAAAGGCCTGACTCCCTC	[21]
		R	TGCCAGTAACAAAGCGAACC	
UBE20	Ubiquitin-conjugating enzyme E2O	F	AGCATCCCCCTTTCCATCAA	Designed in house
		R	CAACCCTGTCTCCTGGCTTA	
**Genes of Interest**				
MDA5	Melanoma Differentiation-Associated protein 5	F	CCACGACCTCTGTGTGCAATT	Designed in house
		R	GCCTGGCCCGCATCTTAT	
RIG-I	Retinoic acid-inducible gene I	F	GTGTATGGAAAACCCTATTTCTTAACT	[22]
		R	GGAGGGTCATACCTGTTGTTTGAT	
RSAD2	Radical S-Adenosyl Methionine Domain Containing 2	F	CCAGCGTCAATTACCACTTCAC	Designed in house
		R	GCAGCACGAAGGAGGTCTTG	
TLR3	Toll-like receptor 3	F	GCAGGCGTATCAGAATTT	Designed in house
		R	CCGACTTTGTTCAATAGC	
TLR7	Toll-like receptor 7	F	CAAATCTTTCAGCTGTGGAAGCACA	Designed in house
		R	CCACTCTCACTGAACCTTCAGAGGC	

Supplementary dataset legends

(Tables/Datasets provided as individual .xlsx data files)

Supplementary Dataset S1. DEGs for poly I:C. List of all genes that were significantly differentially expressed (Benjamini and Hochberg FDR adjusted p-values < 0.05) when comparing the control and the poly I:C treatment group for each timepoint. Log-FC, average log2-expression (CPM), moderated *t*-statistic, raw and adjusted p-value, and log-odds that the gene is differentially expressed for each gene is shown.

Supplementary Dataset S2. DEGs for LPS. List of all genes that were significantly differentially expressed (Benjamini and Hochberg FDR adjusted p-values < 0.05) when comparing the control and the LPS treatment group for each timepoint. Log-FC, average log2-expression (CPM), moderated *t*-statistic, raw and adjusted p-value, and log-odds that the gene is differentially expressed is shown for each gene.

Supplementary Dataset S3. DEGs for *S. aureus*. List of all genes that were significantly differentially expressed (Benjamini and Hochberg FDR adjusted p-values < 0.05) when comparing the control and the *S. aureus* treatment group for each timepoint. Log-FC, average log2-expression (CPM), moderated *t*-statistic, raw and adjusted p-value, and log-odds that the gene is differentially expressed is shown for each gene.

Supplementary Dataset S4. Reciprocal Best Hits. List of the reciprocal best hits (RBHs) to the uncharacterised genes, identified through the reciprocal BLAST search against chicken *Gallus gallus* (GG), turkey *Meleagris gallopavo* (MG), zebra finch *Taeniopygia guttata* (TG), collared flycatcher *Ficedula albicollis* (FA), mouse *Mus musculus* (MM) and human *Homo sapiens* (HS).

Supplementary Dataset S5. Summary GO results Poly I:C. List of all significantly overrepresented biological processes and Reactome pathways for each timepoint in the poly I:C treatment group (FDR < 0.05), retrieved from the PANTHER Overrepresentation Test.

Supplementary Dataset S6. **Summary GO results LPS.** List of all significantly overrepresented biological processes and Reactome pathways for each timepoint in the LPS treatment group (FDR < 0.05), retrieved from the PANTHER Overrepresentation Test.

Supplementary Dataset S7. Fold change and corrected p-value for KEGG pathway genes. Log2 fold change and adjusted p-value (FDR) for each of the three treatments for all characterised genes within each of the seven immune related KEGG pathways available for *Anas platyrhynchos*[16-18]. The data was retrieved as part of our differential gene expression analysis.

References

1 Law, C. W., Alhamdoosh, M., Su, S., Smyth, G. K. & Ritchie, M. E. RNA-seq analysis is easy as 1-2-3 with limma, Glimma and edgeR. *F1000Res* **5**, 1408, doi:10.12688/f1000research.9005.2 (2016).

2 Andrews, S. FastQC: a quality control tool for high throughput sequence data. (2010).

3 Huang, Y. *et al.* The duck genome and transcriptome provide insight into an avian influenza virus reservoir species. *Nat. Genet.* **45**, 776-783, doi:10.1038/ng.2657 (2013).

4 Kim, D., Langmead, B. & Salzberg, S. L. HISAT: a fast spliced aligner with low memory requirements. *Nat. Methods* **12**, 357-360, doi:10.1038/nmeth.3317 (2015).

5 Lawrence, M. *et al.* Software for computing and annotating genomic ranges. *PLoS Comput. Biol.* **9**, e1003118, doi:10.1371/journal.pcbi.1003118 (2013).

6 Pertea, M. *et al.* StringTie enables improved reconstruction of a transcriptome from RNA-seq reads. *Nat. Biotechnol.* **33**, 290-295, doi:10.1038/nbt.3122 (2015).

7 Altschul, S. F., Gish, W., Miller, W., Myers, E. W. & Lipman, D. J. Basic local alignment search tool. *Mol. Biol.* **215**, 403-410, doi:10.1016/S0022-2836(05)80360-2 (1990).

8 Pearson, W. R. An introduction to sequence similarity ("homology") searching. *Curr. Protoc. Bioinformatics*, 3.1. 1-3.1. 8, doi:10.1002/0471250953.bi0301s42 (2013).

9 Kinsella, R. J. *et al.* Ensembl BioMarts: a hub for data retrieval across taxonomic space. *Database* **2011**, bar030-bar030, doi:10.1093/database/bar030 (2011).

10 Robinson, M. D., McCarthy, D. J. & Smyth, G. K. edgeR: a Bioconductor package for differential expression analysis of digital gene expression data. *Bioinformatics* **26**, 139-140, doi:10.1093/bioinformatics/btp616 (2010).

11 McCarthy, D. J., Chen, Y. & Smyth, G. K. Differential expression analysis of multifactor RNA-Seq experiments with respect to biological variation. *Nucleic Acids Res.* **40**, 4288-4297, doi:10.1093/nar/gks042 (2012).

12 Ritchie, M. E. *et al.* Limma powers differential expression analyses for RNA-sequencing and microarray studies. *Nucleic Acids Res.* **43**, e47-e47, doi:10.1093/nar/gkv007 (2015).

13 Smyth, G. K. Linear models and empirical bayes methods for assessing differential expression in microarray experiments. *Stat. Appl. Genet. Mol. Biol.* **3**, 1-25, doi:10.2202/1544-6115.1027 (2004).

14 Benjamini, Y. Discovering the false discovery rate. *J. Royal Stat. Soc.* **72**, 405-416, doi:10.1111/j.1467-9868.2010.00746.x (2010).

15 Thomas, P. D. *et al.* PANTHER: a library of protein families and subfamilies indexed by function. *Genome Res.* **13**, 2129-2141, doi:10.1101/gr.772403 (2003).

16 Kanehisa, M. & Goto, S. KEGG: kyoto encyclopedia of genes and genomes. *Nucleic Acids Res.* **28**, 27-30, doi:10.1093/nar/28.1.27 (2000).

17 Kanehisa, M. Toward understanding the origin and evolution of cellular organisms. *Protein Sci.* **28**, 1947-1951, doi:10.1002/pro.3715 (2019).

18 Kanehisa, M., Furumichi, M., Sato, Y., Ishiguro-Watanabe, M. & Tanabe, M. KEGG: integrating viruses and cellular organisms. *Nucleic Acids Res.* **49**, D545-D551, doi:10.1093/nar/gkaa970 (2021).

19 Rohn, H. *et al.* VANTED v2: a framework for systems biology applications. *BMC Syst. Biol.* **6**, 139, doi:10.1186/1752-0509-6-139 (2012).

20 Junker, A. *et al.* Creating interactive, web-based and data-enriched maps with the Systems Biology Graphical Notation. *Nat. Protoc.* **7**, 579, doi:10.1038/nprot.2012.002 (2012).

21 Chapman, J. R. *et al.* A Panel of Stably Expressed Reference Genes for Real-Time qPCR Gene Expression Studies of Mallards (Anas platyrhynchos). *PloS one* **11**, e0149454, doi:10.1371/journal.pone.0149454 (2016).

22 Barber, M. R., Aldridge, J. R., Webster, R. G. & Magor, K. E. Association of RIG-I with innate immunity of ducks to influenza. *Proc. Natl. Acad. Sci. U.S.A.* **107**, 5913-5918, doi:10.1073/pnas.1001755107 (2010).

23 Bustin, S. A. *et al.* The MIQE guidelines: minimum information for publication of quantitative real-time PCR experiments. *Clin. Chem.* **55**, 611-622, doi:10.1373/clinchem.2008 (2009).
24 Prediger, E. *Designing PCR primers and probes*, <https://eu.idtdna.com/pages/decoded/decoded-articles/pipet-tips/decoded/2013/10/21/designing-pcr-primers-and-probes> (2016).
25 LifeTechnologies. *Real-Time qPCR handbook*, <http://www.gene-quantification.com/real-time-pcr-handbook-life-technologies-update-flr.pdf> (2012).
26 Downey, N. *Interpreting melt curves: An indicator, not a diagnosis*, <https://eu.idtdna.com/pages/decoded/decoded-articles/core-concepts/decoded/2014/01/20/interpreting-melt-curves-an-indicator-not-a-diagnosis> (2016).
27 Rao, X., Huang, X., Zhou, Z. & Lin, X. An improvement of the 2ˆ (–delta delta CT) method for quantitative real-time polymerase chain reaction data analysis. *Biostat. Bioinform. Biomath.* **3**, 71 (2013).
28 Medzhitov, R. Toll-like receptors and innate immunity. *Nat. Rev. Immunol.* **1**, 135, doi:10.1038/35100529 (2001).
29 Takeda, K. & Akira, S. TLR signaling pathways. *Semin. Immunol.* **16**, 3-9, doi:10.1016/j.smim.2003.10.003 (2004).
30 Lemaitre, B., Nicolas, E., Michaut, L., Reichhart, J.-M. & Hoffmann, J. A. The dorsoventral regulatory gene cassette spätzle/Toll/cactus controls the potent antifungal response in *Drosophila* adults. *Cell* **86**, 973-983, doi:10.1016/S0092-8674(00)80172-5 (1996).
31 Medzhitov, R., Preston-Hurlburt, P. & Janeway Jr, C. A. A human homologue of the *Drosophila* Toll protein signals activation of adaptive immunity. *Nature* **388**, 394, doi:10.1038/41131 (1997).
32 Cormican, P. *et al.* The avian Toll-Like receptor pathway—Subtle differences amidst general conformity. *Dev. Comp. Immunol.* **33**, 967-973, doi:10.1016/j.dci.2009.04.001 (2009).
33 Temperley, N. D., Berlin, S., Paton, I. R., Griffin, D. K. & Burt, D. W. Evolution of the chicken Toll-like receptor gene family: A story of gene gain and gene loss. *BMC Genomics* **9**, 62, doi:10.1186/1471-2164-9-62 (2008).
34 Brownlie, R. & Allan, B. Avian toll-like receptors. *Cell Tissue Res.* **343**, 121-130, doi:10.1007/s00441-010-1026-0 (2011).
35 Boyd, A. C. *et al.* TLR15 is unique to avian and reptilian lineages and recognizes a yeast-derived agonist. *J. Immunol.*, 1101790, doi:10.4049/jimmunol.1101790 (2012).
36 Matsumoto, M. & Seya, T. TLR3: Interferon induction by double-stranded RNA including poly(I:C). *Adv. Drug Deliv. Rev.* **60**, 805-812, doi:10.1016/j.addr.2007.11.005 (2008).
37 Zhang, M. *et al.* Molecular cloning of Peking duck Toll-like receptor 3 (duTLR3) gene and its responses to reovirus infection. *Virol. J.* **12**, 207, doi:10.1186/s12985-015-0434-x (2015).
38 Jiao, P. *et al.* Molecular cloning, characterization, and expression analysis of the Muscovy duck Toll-like receptor 3 (MdTLR3) gene. *Poult. Sci.* **91**, 2475-2481, doi:10.3382/ps.2012-02394 (2012).
39 Kang, Y. *et al.* Host innate immune responses of ducks infected with Newcastle disease viruses of different pathogenicities. *Front Microbiol.* **6**, 1283, doi:10.3389/fmicb.2015.01283 (2015).
40 Li, N. *et al.* Pathogenicity of duck plague and innate immune responses of the Cherry Valley ducks to duck plague virus. *Sci. Rep.* **6**, 32183, doi:10.1038/srep32183 (2016).
41 Erridge, C. & Spickett, C. M. Oxidised phospholipid regulation of Toll-like receptor signalling. *Redox Report* **12**, 76-80, doi:10.1179/135100007X162121 (2007).
42 Wang, H. & Jiang, C. Avian influenza H5N1: an update on molecular pathogenesis. *Sci. China C Life Sci.* **52**, 459-463, doi:10.1007/s11427-009-0059-07 (2009).
43 Cornelissen, J. B. W. J., Post, J., Peeters, B., Vervelde, L. & Rebel, J. M. J. Differential innate responses of chickens and ducks to low-pathogenic avian influenza. *Avian Pathol.* **41**, 519-529, doi:10.1080/03079457.2012.732691 (2012).

44 Song, C. *et al.* Effect of age on the pathogenesis of DHV-1 in Pekin ducks and on the innate immune responses of ducks to infection. *Arch. Virol.* **159**, 905-914, doi:10.1007/s00705-013-1900-7 (2014).
45 Beutler, B. Innate immunity: an overview. *Mol. Immunol.* **40**, 845-859, doi:10.1016/j.molimm.2003.10.005 (2004).
46 Schaeffer, H. J. & Weber, M. J. Mitogen-activated protein kinases: specific messages from ubiquitous messengers. *Mol. Cell. Biol.* **19**, 2435-2444, doi:10.1128/MCB.19.4.2435 (1999).
47 Pearson, G. *et al.* Mitogen-activated protein (MAP) kinase pathways: regulation and physiological functions. *Endocr. Rev.* **22**, 153-183, doi:10.1210/edrv.22.2.0428 (2001).
48 Li, Q. & Verma, I. M. NF-κB regulation in the immune system. *Nat. Rev. Immunol.* **2**, 725, doi:10.1038/nri910 (2002).
49 Baeuerle, P. A. & Baltimore, D. I kappa B: a specific inhibitor of the NF-kappa B transcription factor. *Science* **242**, 540-546, doi:10.1126/science.3140380 (1988).
50 Kirschning, C. & Schumann, R. in *Toll-like receptor family members and their ligands* 121-144 (Springer, 2002).
51 Fukui, A. *et al.* Molecular cloning and functional characterization of chicken toll-like receptors A single chicken toll covers multiple molecular patterns. *J. Biol. Chem.* **276**, 47143-47149, doi:10.1074/jbc.M103902200 (2001).
52 Marais, M., Gugushe, N., Maloney, S. & Gray, D. Body temperature responses of Pekin ducks (*Anas platyrhynchos domesticus*) exposed to different pathogens. *Poult. Sci.* **90**, 1234-1238, doi:10.3382/ps.2011-01389 (2011).
53 Fortier, M.-E. *et al.* The viral mimic, polyinosinic:polycytidylic acid, induces fever in rats via an interleukin-1-dependent mechanism. *Am. J. Physiol. Regul. Integr. Comp. Physiol.* **287**, R759-R766, doi:10.1152/ajpregu.00293.2004 (2004).
54 Homan, E. R., Zendzian, R. P., Schott, L. D., Levy, H. B. & Adamson, R. H. Studies on poly I: C toxicity in experimental animals. *Toxicol. Appl. Pharmacol.* **23**, 579-588, doi:10.1016/0041-008X(72)90098-1 (1972).
55 Kimura, M. *et al.* Comparison of acute phase responses induced in rabbits by lipopolysaccharide and double-stranded RNA. *Am. J. Physiol. Regul. Integr. Comp. Physiol.* **36**, R1596, doi:10.1152/ajpregu.1994.267.6.R1596 (1994).
56 Alexander, C. & Rietschel, E. T. Invited review: bacterial lipopolysaccharides and innate immunity. *J. Endotoxin Res.* **7**, 167-202, doi:10.1177/09680519010070030101 (2001).
57 Owen-Ashley, N. T., Turner, M., Hahn, T. P. & Wingfield, J. C. Hormonal, behavioral, and thermoregulatory responses to bacterial lipopolysaccharide in captive and free-living white-crowned sparrows *(Zonotrichia leucophrys gambelii*). *Horm. Behav.* **49**, 15-29, doi:10.1016/j.yhbeh.2005.04.009 (2006).
58 Owen-Ashley, N. T. & Wingfield, J. C. Seasonal modulation of sickness behavior in free-living northwestern song sparrows (*Melospiza melodia morphna*). *J. Exp. Biol.* **209**, 3062-3070, doi:10.1242/jeb.02371 (2006).
59 Matson, K. D., Ricklefs, R. E. & Klasing, K. C. A hemolysis–hemagglutination assay for characterizing constitutive innate humoral immunity in wild and domestic birds. *Dev. Comp. Immunol.* **29**, 275-286, doi:10.1016/j.dci.2004.07.006 (2005).
60 Luker, F. I., Mitchell, D. & Laburn, H. P. Fever and motor activity in rats following day and night injections of *Staphylococcus aureus* cell walls. *Am. J. Physiol. Regul. Integr. Comp. Physiol.* **279**, R610-R616, doi:10.1152/ajpregu.2000.279.2.R610 (2000).
61 Korte, S. M., Ruesink, W. & Blokhuis, H. J. Heart Rate Variability During Manual Restraint in Chicks From High- and Low-Feather Pecking Lines of Laying Hens. *Physiol. Behav.* **65**, 649-652, doi:10.1016/S0031-9384(98)00206-6 (1998).
62 Cyr, N. E., Dickens, M. J. & Romero, L. M. Heart rate and heart-rate variability responses to acute and chronic stress in a wild-caught passerine bird. *Physiol. Biochem. Zool.* **82**, 332-344, doi:10.1086/589839 (2009).

63 Gray, D. A., Maloney, S. K. & Kamerman, P. R. Restraint increases afebrile body temperature but attenuates fever in Pekin ducks (*Anas platyrhynchos*). *Am. J. Physiol. Regul. Integr. Comp. Physiol.* **294**, R1666-R1671, doi:10.1152/ajpregu.00865.2007 (2008).

64 Cabanac, M. & Aizawa, S. Fever and tachycardia in a bird (*Gallus domesticus*) after simple handling. *Physiol. Behav.* **69**, 541-545, doi:10.1016/S0031-9384(00)00227-4 (2000).

65 Cabanac, A. J. & Guillemette, M. M. Temperature and heart rate as stress indicators of handled common eider. *Physiol. Behav.* **74**, 475-479, doi:10.1016/S0031-9384(01)00586-8 (2001).

66 Skwarska, J. Variation of heterophil-to-lymphocyte ratio in the Great Tit Parus major—a review. *Acta Ornithol.* **53**, 103-114, doi:10.3161/00016454AO2018.53.2.001 (2019).

67 Sepp, T., Sild, E. & Horak, P. Hematological condition indexes in greenfinches: effects of captivity and diurnal variation. *Physiol. Biochem. Zool.* **83**, 276-282, doi:10.1086/648580 (2010).

68 Roshier, D. A. & Asmus, M. W. Use of satellite telemetry on small-bodied waterfowl in Australia. *Mar. Freshw. Res.* **60**, 299-305, doi:10.1071/MF08152 (2009).

69 D'alecy, L. & Kluger, M. Avian febrile response. *J. Physiol.* **253**, 223 (1975).

70 Korner, P., Sauter, A., Fiedler, W. & Jenni, L. Variable allocation of activity to daylight and night in the mallard. *Anim. Behav.* **115**, 69-79, doi:10.1016/j.anbehav.2016.02.026 (2016).

71 R Core Team. (2014).

72 Wood, S. N. Fast stable restricted maximum likelihood and marginal likelihood estimation of semiparametric generalized linear models. *J. R. Stat. Soc. Series B Stat. Methodol.* **73**, 3-36, doi:10.1111/j.1467-9868.2010.00749.x (2011).

73 Pinheiro, J., Bates, D., DebRoy, S., Sarkar, D. & Team, R. C. nlme: Linear and nonlinear mixed effects models. *R package version* **3**, 96 (2009).

74 Box, G. E., Jenkins, G. M., Reinsel, G. C. & Ljung, G. M. *Time series analysis: forecasting and control*. 712 (John Wiley & Sons, 2015).

75 Gelman, A. *et al. Bayesian data analysis*. Vol. 2 (CRC press, 2014).

76 Pierre, R. V. Peripheral blood film review: the demise of the eyecount leukocyte differential. *Clin. Lab. Med.* **22**, 279-297, doi:10.1016/S0272-2712(03)00075-1 (2002).

77 Seliger, C. *et al.* A rapid high-precision flow cytometry based technique for total white blood cell counting in chickens. *Vet. Immunol. Immunopathol.* **145**, 86-99, doi:10.1016/j.vetimm.2011.10.010 (2012).

78 Pendl, H. & Samour, J. in *Avian Medicine* Vol. 3 (ed Jamie Samour) 77-99 (Elsevier, 2016).

79 Reauz, B., Scope, A., Hauska, H. & Vasicek, L. Vergleich hämatologischer Untersuchungsmethoden bei Vögeln. *Tierarztl. Prax. Ausg. K Kleintiere Heimtiere* **27**, 65-73 (1999).

80 Spiegelhalter, D., Thomas, A., Best, N. & Lunn, D. WinBUGS user manual. (2003).

81 Sturtz, S., Ligges, U. & Gelman, A. R2WinBUGS: a package for running WinBUGS from R. *J. Stat. Softw.* **12**, 1-16 (2005).

82 Brooks, S. & Gelman, A. Some issues for monitoring convergence of iterative simulations. *Comput. Sci. Stat.*, 30-36 (1998).

83 Blyth, G. A., Chan, W. F., Webster, R. G. & Magor, K. E. Duck IFITM3 mediates restriction of influenza viruses. *J. Virol.* **90**, 103-116, doi:10.1128/JVI.01593-15 (2016).
